# Conserved allomorphs of MR1 drive the specificity of MR1-restricted TCRs

**DOI:** 10.3389/fonc.2024.1419528

**Published:** 2024-10-03

**Authors:** Terri V. Cornforth, Nathifa Moyo, Suzanne Cole, Emily P. S. Lam, Tatiana Lobry, Ron Wolchinsky, Angharad Lloyd, Katarzyna Ward, Eleanor M. Denham, Giulia Masi, Phyllis Tea Qing Yun, Colin Moore, Selsabil Dhaouadi, Gurdyal S. Besra, Natacha Veerapen, Patricia T. Illing, Julian P. Vivian, Jeremy M. Raynes, Jérôme Le Nours, Anthony W. Purcell, Samit Kundu, Jonathan D. Silk, Luke Williams, Sophie Papa, Jamie Rossjohn, Duncan Howie, Joseph Dukes

**Affiliations:** ^1^ Enara Bio Ltd., Oxford, United Kingdom; ^2^ Institute of Microbiology and Infection, School of Biosciences, University of Birmingham, Birmingham, United Kingdom; ^3^ Infection and Immunity Program and Department of Biochemistry and Molecular Biology, Biomedicine Discovery Institute, Monash University, Clayton, VIC, Australia; ^4^ School of Cancer and Pharmaceutical Sciences, King’s College London, Guy’s Hospital, London, United Kingdom; ^5^ Institute of Infection and Immunity, Cardiff University, School of Medicine, Cardiff, United Kingdom

**Keywords:** MR1, alloreactive, T-cell, (TCR) T-cell receptor, cancer

## Abstract

**Background:**

Major histocompatibility complex class-1-related protein (MR1), unlike human leukocyte antigen (HLA) class-1, was until recently considered to be monomorphic. MR1 presents metabolites in the context of host responses to bacterial infection. MR1-restricted TCRs specific to tumor cells have been described, raising interest in their potential therapeutic application for cancer treatment. The diversity of MR1-ligand biology has broadened with the observation that single nucleotide variants (SNVs) exist within MR1 and that allelic variants can impact host immunity.

**Methods:**

The TCR from a MR1-restricted T-cell clone, MC.7.G5, with reported cancer specificity and pan-cancer activity, was cloned and expressed in Jurkat E6.1 TCRαβ− β2M− CD8+ NF-κB:CFP NFAT:eGFP AP-1:mCherry cells or in human donor T cells. Functional activity of 7G5.TCR-T was demonstrated using cytotoxicity assays and by measuring cytokine release after co-culture with cancer cell lines with or without loading of previously described MR1 ligands. MR1 allele sequencing was undertaken after the amplification of the MR1 gene region by PCR. *In vivo* studies were undertaken at Labcorp Drug Development (Ann Arbor, MI, USA) or Epistem Ltd (Manchester, UK).

**Results:**

The TCR cloned from MC.7.G5 retained MR1-restricted functional cytotoxicity as 7G5.TCR-T. However, activity was not pan-cancer, as initially reported with the clone MC.7.G5. Recognition was restricted to cells expressing a SNV of MR1 (MR1*04) and was not cancer-specific. 7G5.TCR-T and 7G5-like TCR-T cells reacted to both cancer and healthy cells endogenously expressing MR1*04 SNVs, which encode R9H and H17R substitutions. This allelic specificity could be overcome by expressing supraphysiological levels of the wild-type MR1 (MR1*01) in cell lines.

**Conclusions:**

Healthy individuals harbor T cells reactive to MR1 variants displaying self-ligands expressed in cancer and benign tissues. Described “cancer-specific” MR1-restricted TCRs need further validation, covering conserved allomorphs of MR1. Ligands require identification to ensure targeting MR1 is restricted to those specific to cancer and not normal tissues. For the wider field of immunology and transplant biology, the observation that MR1*04 may behave as an alloantigen warrants further study.

## Introduction

The MHC class 1-related protein 1 (MR1) is one of several nonclassical human leukocyte antigen (HLA) molecules (1–8). Functionally, MR1 presents antigens from folate and riboflavin-derived metabolites to the immune system, some of which are derived from bacteria or yeast. Unlike the classical polymorphic HLA class 1 molecules, which present peptide antigens (pHLA), MR1 was widely considered to be monomorphic. However, in 2021, Rozemuller et al. identified five distinct MR1 allele group variants in a series of 56 DNA samples taken from cells with diversity in HLA (9). They adopted MR1*01 as the nomenclature of the wild-type allele with MR1*02, the most common variant at 21% in their analysis, demonstrating a single nucleotide polymorphism (SNP) at H17R in the parent protein. MR1*04 has both an R9H and H17R polymorphism and has a heterozygous frequency of approximately one in 100 Caucasians.

Structural studies of MR1 molecules demonstrate a similar overall architecture to HLA class 1 molecules, formed of a heavy chain composed of α1 and α2 helices forming the binding groove and an α3 domain noncovalently bound to β2m (10, 11). The binding groove of MR1 has an A’ and an F’ pocket. The A’ pocket binds ligands and is lined with aromatic and basic residues. These residues create an environment permissive for the binding of microbially derived vitamin B12 metabolites and restrict the space available for binding to peptides (2, 10, 11). The two basic residues in the A’ pocket, R9 and K43, are critical for interaction with ligands. R9 is conserved across mammalian species and interacts directly with ligands (12). K43 is essential for the formation of covalent Schiff bonds with many pyrimidine-based ligands (1, 12–14).

Many aspects of MR1 biology have been revealed by studies of a unique class of T cells, known as mucosal-associated invariant T (MAIT) cells, which recognize ligands derived from bacterial metabolites bound to MR1 using a limited (invariant) set of αβ (TCRs) (3, 15–20). The nature of the ligands presented by MR1 to MAIT cells has been the subject of numerous investigations (1, 21–23). Interestingly, the antigens and TCRs are conserved sufficiently such that there is cross-reactivity between human and murine-derived MR1s and MAIT cells. The semi-invariant nature of MAIT TCRs and the unique developmental pathway instructed by PLZF expression indicate that this class of T cells operates at the interface between adaptive and innate immunity.

A distinct subset of MR1-restricted T cells (MR1T) has been identified with the apparent ability to specifically recognize and kill cancer cells (24–26). The MR1 ligands recognized by cancer-specific MR1T remain poorly understood but appear distinct from those of MAIT cells, as shown by the lack of enhanced reactivity on the addition of 5-OP-RU (26) and increased reactivity by MR1T to nucleobase adducts (27). Within the MR1T cell family, there are patterns of ligand preference illustrated by a differential dependence for recognition on the K43 residue in the MR1-binding groove (25). Isolation and study of TCRs from putative MR1T present an attractive potential source of novel cancer therapies. Targeting peptides presented by specific HLA class 1 molecules (e.g., HLA-A*02:01, HLA-B*08:01, HLA-C*07:01, etc.) precludes the broad utility of a TCR-based therapy across all patients as they must be used in appropriately targeted subsets of the human population with the matching HLA type. The reported monomorphic nature of MR1 raised the possibility that therapies directed toward MR1-displayed antigens have the potential to be effective across the entire human population. However, recent reverse translation from a patient with a pervasive and unusual chronic infective phenotype resulted in the identification of a single nucleotide polymorphism in the patient’s MR1, for which they were homozygous (12). The point mutation was identified at R9H of the mature MR1 protein, within the antigen-binding groove of MR1 (26, 27).

The investigators observed that MAIT cells were completely absent in the patient’s circulating peripheral blood mononuclear cells (PBMC), demonstrating functional outcomes of polymorphisms in MR1.

We undertook to express TCRs isolated from MR1T clones in polyclonal human T cells to test the hypothesis that these TCRs could have pan-cancer therapeutic utility. Validation of our approach led to the understanding that conserved SNPs present in MR1 can drive T-cell activation, in a highly MR1*04-specific manner, that is not exclusive to tumor cells (9, 12). Collectively, these data highlight the need for a deeper understanding of MR1 biology in the context of cancer and raise the possibility that MR1 polymorphism may need to be considered in the context of allotransplantation and graft vs. host disease (GvHD).

## Materials and methods

### Cell lines and cell culture

Cancer cell lines were cultured according to the manufacturer’s instructions.

### Blood derived cells

Whole blood was sourced from research donors via Cambridge Bioscience, under local ethical review panel guidelines. PBMCs were isolated from whole blood using lymphoprep and leukosep tubes according to the manufacturer’s instructions. PBMCs were used as starting material to isolate B cells (CD19+ microbeads, Miltenyi Biotec, Bergisch Gladbach, Germany) and monocytes (CD14+ microbeads, Miltenyi Biotec) following the manufacturers’ protocols.

### ELISA assays

All co-culture assays were carried out in RPMI (Thermo Fisher Scientific, Waltham, MA, USA Cat. 21875091 or Sigma-Aldrich, Cat. R8758) containing 10% fetal bovine serum (Thermo Fisher Scientific, Cat. 16140071). Target cells were plated in flat-bottomed 96-well plates, and T cells were thawed and rested for 2 h prior to plating. Target cell numbers, T-cell numbers, and effector-to-target ratios can be found in relevant figure legends. Target and T-cell co-cultures were incubated at 37°C in 5% CO_2_. Co-culture durations can be found in relevant figure legends. Supernatants were collected from effector-target cell co-cultures and analyzed for IFN-γ using either ELISA MAX Deluxe Human IFN-γ kit (BioLegend, San Diego, CA, USA) or Human IFN-γ DuoSet ELISA (R&D Systems) and for granzyme B using human Granzyme B DuoSet ELISA (R&D Systems, Minneapolis, MN, USA). All ELISAs were performed as per the manufacturer’s instructions. ELISAs were read on a Mini ELISA plate reader from BioLegend.

### ELISpot assay

IFN-γ ELISpot assays were performed using the Human IFN-γ (ALP) kit (Mabtech, Nacka Strand, Sweden Cat. No. 3420-4APW-10). Briefly, anti-CD3 mAb (2µg/ml) was added to the required wells of the anti-IFN-γ (D1K) mAb precoated plate for 30 min. Dulbecco's Phosphate-Buffered Saline (DPBS) was added to all remaining wells. Wells were then washed five times with DPBS before subsequent blocking of plates with RPMI (Thermo Fisher Scientific, Cat. 21875091 or Sigma-Aldrich, Cat. R8758) containing 10% fetal bovine serum (Thermo Fisher Scientific, Cat. 16140071). Plates were incubated for 1 h before washing five times with DPBS. Target cells were then plated at the appropriate density and incubated for 2 h before effector cells were added for the required E:T ratios. Following overnight activation, the cells were washed from the plate and stained with IFN-γ (7-B6-1-biotin) detection antibody (1 µg/ml) and streptavidin-ALP (1:1,000 dilution). BCIP/NBT-Plus substrate solution was added until spots emerged, and color development was stopped by extensive washing in tap water. Plates were dried before being imaged and counted using an ELISpot plate reader (CTL S6 Ultra V) and ImmunoSpot SC Suite software.

### Ac-6FP and anti-MR1 cell treatment

Target cells were plated at 20,000 cells per well in a flat bottom 96-well plate and allowed to attach for 2 h. Following this, cells were preincubated with 10 μg/ml anti-MR1 clone 26.5 (BioLegend) or 100 μM Ac-6-FP for 4 h before adding 60,000 T cells and incubating for an additional 18 h at 37°C in 5% CO_2_.

### Flow cytometry

Adherent cells were harvested from plates using TrypLE Express (Thermo Fisher Scientific) and transferred to a 96-well round-bottom plate for staining. Cells were stained with Zombie Violet Viability Dye (BioLegend) in PBS for 10 min at 4°C, followed by anti-MR1 APC (clone 26.5, BioLegend) or isotype control (MOPC-173, BioLegend) in FACS buffer for 30 min at 4°C. Cells were washed twice, resuspended, and acquired on a (Beckman Coulter, Indianapolis, IN, USA) Cytoflex-S flow cytometer. The data were analyzed using FlowJo software.

### TCR sequences

Genes encoding the full-length TCR-α and TCR-β chains linked by a 2A-furin sequence for AVA34 DGB129 and TC5A87 according to the sequences in patent WO2021144475A1, 759s, A4, and C1 from patent WO2023148494 were synthesized and cloned into the vector pSF-LV-EF1a (Oxgene, Oxford, UK) using the GeneArt service (Thermo Fisher Scientific).

### Transfection of HEK293T Lenti-X cells and virus concentration

HEK293T Lenti-X cells were transfected using PEIpro (Polyplus, Illkirch-Graffenstaden, France) as per the manufacturer’s instructions with a lentivector encoding the appropriate T-cell receptor or MR1 construct alongside pREV.Kan, pGagPol.Kan, and pVSVG.Kan packaging vectors (Aldevron, Fargo, ND, USA). After 72 h, virus-containing cell supernatants were concentrated using Amicon Ultra-15 Centrifugal Filter Units (Merck Millipore, Burlington, MA, USA) and exchanged into TexMACS media (Miltenyi Biotec).

### Cell line transduction with MR1

Cell lines to be transduced were thawed and rested overnight in RPMI (Thermo Fisher Scientific, Cat. 21875091 or Sigma-Aldrich, Cat. R8758) containing 10% fetal bovine serum (Thermo Fisher Scientific, Cat. 16140071) at 37°C and 5% CO_2_. After resting, cells were harvested, counted, and plated at 300,000 cells per well in a six-well plate. Cells were then transduced to express B2M-MR1 using concentrated supernatant from transfected HEK293T, as described above, and polybrene (dilution 1:1000). Efficiency was analyzed 6–7 days posttransduction using flow cytometry.

### Lentiviral transduction of primary T cells and Jurkat cells

Jurkat E6.1 TCRαβ− β2M− CD8+ NF-κB:CFP NFAT:eGFP AP-1:mCherry cells (a kind gift from Peter Steinberger, Medical University of Vienna) were transduced to express a TCR using concentrated supernatant from transfected HEK293T cells and Polybrene. Transduction efficiency was analyzed 3–4 days following transduction using flow cytometry. CD3+ T cells were purified from human peripheral blood LeukoPaks (HemaCare, Los Angeles, CA, USA) using Cell Therapy Systems CD3/CD28 Dynabeads (Thermo Fisher Scientific). Positively selected CD3+ cells (15 × 10^6^) and Dynabeads in TexMACS (Miltenyi Biotech) 5% human AB serum (Life Science Production, Sandy, UK Cat. S-104B-HI-US) 20 ng/ml IL-2 (PeproTech, London, UK Cat. 200-02) were seeded into 10M G-Rex systems (Wilson Wolf, St Paul, MN, USA) and incubated overnight at 37°C in 5% CO_2_. CD3+ cells were transduced to express a TCR with or without CD8 or mock transduced using concentrated supernatant from transfected HEK293T cells and LentiBOOST (Sirion Bio, Graefelfing, Germany). For some *in vivo* experiments, clustered regularly interspaced short palindromic repeats (CRISPR)/CRISPR-associated protein-9 (Cas9)-mediated knockout of both TCR-β constant regions (*trbc1* and *trbc2*) was performed on TCR-T cells. A pLentiCRISPR v2 plasmid-encoding four guide RNAs (gRNAs) targeting the first exon of the *trbc* gene segments was used (Addgene, Watertown, MA, USA Plasmid No. 52961, and inserts kindly provided by Andy Sewell, Cardiff University). pLentiCRISPR v2 plasmid encodes SpCas9 protein and a puromycin-resistance marker gene (puromycin *N*-acetyltransferase [pac]). The sequence alignments of gRNAs are summarized in Legut et al. (28). Lentiviral particles were generated by calcium chloride transfection of HEK 293T cells. CRISPR/Cas9 vectors were cotransfected with packaging and envelope plasmids pMD2.G and psPAX2. Lentiviral particles were concentrated by ultracentrifugation prior to the transduction of T cells.

Following a further 24 h, fresh TexMACS 5% human AB serum 20 ng/ml IL-2 was added to G-Rex systems, where the cells were transduced with CRISPR Cas9 puromycin (Thermo Fisher Scientific) at a final concentration of 1 µg/ml. CD3+ cells were harvested, magnetic beads removed, and frozen for storage 10 days following transduction and expansion.

### Generation of the JRT76 β2m^ko^ cell line via CRISPR/Cas9

Jurkat clone 76 (JRT76) cells (29), a TCRα/β-negative derivative of the Jurkat E6.1 cell line (ATCC TIB-152™), were kindly provided by Prof Mirjam H M Heemskerk, Leiden University. Knockout of β2m was performed using the pLentiCRISPR v2 CRISPR/Cas9 system (30) using a pLentiCRISPR v2 plasmid encoding the β2m-targeting guide RNA (gRNA): GAGTAGCGCGAGCACAGCTA (Genscript, Piscataway, NJ, USA). Lentiviral particles were packaged in HEK293T cells (ATCC: CRL-3216™) by cotransfection of pLentiCRISPR v2-β2m-gRNA with pRSV-rev (Addgene Plasmid No. 12253), pMDLg/pRRE (Addgene Plasmid No. 12251), and pMD2.G (Plasmid No. 12259) plasmids, which were gifts from Didier Trono, obtained through Addgene. Transfections were performed by complexing plasmids with FuGENE® 6 Transfection Reagent (Promega, Madison, WI, USA) in OptiMEM media (Thermo Fisher Scientific) before pipetting dropwise onto plated HEK293T cells in D10 culture media: Dulbecco’s modified Eagle’s medium (DMEM) supplemented with 10% heat-inactivated fetal calf serum (FCS), 2 mM GlutaMAX™, 100 units/ml penicillin, and 100 µg/ml streptomycin (all Thermo Fisher Scientific). Transfections were incubated for ~16 h, after which cells were replenished with fresh D10 media. Viral particle-containing media was harvested after a further 24 and 48 h and pooled.

Target JRT76 cells were transduced by culturing 6.0 × 10^5^ cells in neat media containing harvested lentivirus. After ~16 h of incubation with lentivirus, cells were replenished with enriched-R10 media: RPMI supplemented with 10% heat-inactivated FCS, 2 mM GlutaMAX™, 100 units/ml penicillin, 100 µg/ml streptomycin, 0.02 M HEPES, 1 mM nonessential amino acids, and 1 mM sodium pyruvate (all Thermo Fisher Scientific). After 72 h in culture, transduced cells underwent antibiotic selection via the addition of 0.5 μg/ml puromycin to culture media for 7 days. Transduction/knockout efficiency was assessed by surface HLA-A,B,C expression; staining cells with W6/32 clone hybridoma supernatant, which was detected via a Goat antimouse-Cy5 secondary (Thermo Fisher Scientific). Subsequent staining was analyzed via flow cytometry using a BD® LSR II Flow Cytometer. From the parent transduced line, a subclonal population of JRT76 β2m-knockout cells that exhibited a complete absence of HLA-A,B,C expression was achieved via limit dilution cloning.

### Activation assays with Jurkat.β2m^ko^.7G5

Jurkat.β2m^ko^.7G5 cells were coincubated at a 1:1 ratio with C1R derivatives (10^5^:10^5^) in 96-well U-bottom plate overnight at 37°C in 5% CO_2_ in RF10. Cells were stained for viability (LIVE/DEAD™ fixable Aqua stain, Thermo Fisher Scientific) and surface expression of CD3 (CD3 PE-Cy7, clone SK7, BD) and CD69 (CD69 APC, clone L78, BD) in PBS and fixed with 1% paraformaldehyde in PBS prior to flow cytometry acquisition. Flow cytometry acquisition was performed on an BD LSRII flow cytometer run with BD FACSDiva software (FlowCore, Monash University) and analyzed using FlowJo™ software. Cells were gated on FSC-A vs. SSC-A, FSC-A vs. FSC-H, GFP vs. Live/Dead, GFP vs. CD3, and the %CD69^hi^ cells extracted (**Supplementary Figure S5**). In parallel, on the same day, C1R derivatives were stained for MR1 expression using 8F2.F9 hybridoma supernatant, followed by antimouse IgG-PE (Goat F(ab′) 2 antimouse IgG (H + L) human ads-PE, Southern Biotech, Birmingham, AL, USA). Cells were gated based on FSC-A vs. SSC-A and FSC-A vs. FSC-H, and the median fluorescence intensity for PE was then extracted (**Supplementary Figure S4**). The data were plotted using GraphPad Prism (GraphPad Software, San Diego, CA, USA).

### Viral copy number analysis

The virus copy number (VCN) was calculated from the concentrations of a viral gene (Psi) and a reference gene (RPP30) measured from the same sample. Genomic DNA was extracted using a Maxwell RSC Instrument (Promega, USA) following the manufacturer’s protocol. Extracted DNA was digested using an *Eco*RI HF Restriction Enzyme (New England Biolabs (NEB), Ipswich, MA, USA). The concentrations of Psi and RPP30 were measured in a QIAcuity Digital PCR instrument (Qiagen, Hilden, Germany) following the manufacturer’s protocol. The following controls were included in each experiment: positive controls with known VCN and no-template controls. The PCR program was 95°C for 2 min of initial heat inactivation, followed by 40 cycles of 15 s of denaturation at 95°C and 30 s combined annealing/extension at 60°C. Primers, probes, positive controls, and buffers were purchased from Integrated DNA Technologies (Integrated DNA Technologies (IDT), Coralville, IA, USA) unless otherwise stated.

Forward primer, Psi: CAG GAC TCG GCT TGC TGA AG

Reserve primer, Psi: GCA CCC ATC TCT CTC CTT CTA GC

Probe, Psi:/56-FAM/TT TTG GCG T/ZEN/A CTC ACC AG/3IABkFQ/

Forward primer, RPP30: AGTGACTGATGCAGGACATTAC

Reserve primer, RPP30: CAGGGCAGAAGAGGCAAATA

Probe, RPP30:/5HEX/AC GCT GTG T/ZEN/G TGG ATT TCT CCT GA/3IABkFQ/

### Cytotoxicity assays

All cytotoxicity assays were performed using the xCELLigence RTCA MP analyzer and 96-well PET E-plates (Agilent Technologies, Santa Clara, CA, USA). Assays were carried out in RPMI (Thermo Fisher Scientific, Cat. 21875091 or Sigma-Aldrich, Cat. R8758) containing 10% fetal bovine serum (Thermo Fisher Scientific, Cat. 16140071). Prior to target cell seeding background measures were taken using assay media only. Target cells were then seeded at a density predetermined to be optimal for each cell line. Plates were incubated overnight at 37°C in 5% CO_2_ on the xCELLigence, with cell index being measured every 15 min. Effector T cells were thawed and prepared as described for ELISA assays and seeded into E-Plates at a ratio of 5:1 T cells:target cells per well. E-Plates were incubated for a further 48 h at 37°C in 5% CO_2_ in the xCELLigence with cell index being measured every 5 min. Using the analyzer software, raw cell index values were converted to percentage cytolysis using full lysis (target cells incubated overnight in E-Plates before the addition of Tween-20 to a final concentration of 0.25%) and target cell-only controls and normalized to the time point before T-cell addition.

### Co-culture of TCR-expressing Jurkat cells and ligand-loaded C1R cells

C1R or C1R cells over-expressing MR1*01 (C1R.MR1, a kind gift from Andrew Sewell, University of Cardiff) were stained with CellTrace Violet (Thermo Fisher Scientific) as per the manufacturer’s instructions. Cells were then incubated in 96-well flat-bottom plates with M_3_ADE, ONEdC, or ONEdG (a kind gift from Prof Gurdyal Besra, University of Birmingham) to a final concentration of 10 µM or 20 µM in RPMI (Thermo Fisher Scientific, Cat. 21875091 or Sigma-Aldrich, Cat. R8758) containing 10% fetal bovine serum (Thermo Fisher Scientific, Cat. 16140071), vehicle only (0.2% DMSO in complete media), or complete media only, for 5 h at 37°C in 5% CO_2_. Jurkat cells (5E4 cells) transduced with one of eight TCRs, or nontransduced, were added to the C1R-containing wells at a 1:1 ratio and incubated for 24 h at 37°C in 5% CO_2_. C1R-only samples were stained with Zombie NIR Fixable Viability Kit (BioLegend). Samples were analyzed on CytoFLEX S (Beckman Coulter). Events were gated on FSC-H vs. SSC-H, FSC-H vs. FSC-W, and Zombie NIR negative, and then the median fluorescence intensity in the PE channel was extracted. Co-culture samples were stained with Zombie Violet Fixable Viability Kit (BioLegend) followed by anti-CD69 APC antibody (FN50, BioLegend). Samples were analyzed on CytoFLEX S (Beckman Coulter). Events were gated on FSC-H vs. SSC-H, FSC-H vs. FSC-W, Zombie Violet/CellTrace Violet negative, and then the median fluorescence intensity in the APC channel was extracted.

Flow cytometry data were analyzed using FlowJo. Other data were analyzed using GraphPad Prism.

### Sequencing of MR1 alleles

Genomic DNA from the MC.7.G5 clone was extracted using the DNeasy Blood and Tissue Kit (Qiagen) and from the blood by using either the DNeasy Blood and Tissue Kit (Qiagen) or the Maxwell^®^ RSC Blood DNA Kit. The MR1 locus was amplified from the DNA using PCR with Q5 High-Fidelity DNA Polymerase (New England Biolabs, Ipswich, MA, USA) following the manufacturer’s instructions. To amplify the region of the MR1 gene encoding the R9 and H17 residues, the following primers were used: OP71_forward_genomic_R9Hmut 5′-CACACGTGCACACACAGAGGTG and OP72_reverse_R9Hmut 5′-GGACAGTCCAGAAGATGCACAGG. PCR products were checked by running on a 1% SyberSAFE agarose gel with a 1-kb DNA ladder (Invitrogen, Waltham, MA, USA). PCR products of successful reactions were purified and sequenced by Source BioScience using following primers: OP80_forward_Exon2_seq 5′-GAGCTCTTACGTCCTGTCCAGG, OP81_reverse_Exon2_seq 5′-GCTACAGCAGGTGCAATTCAGC, OP82_reverse_Exon2_seq 5′-GCGAGGTTCTCTGCCATCC, OP83_forward_Exon2_seq 5′-CAGTGTCACTCGGCAGAAGG, OP69_genomic primer 2F 5′-GAAGAAGGCTGCGTCATCAG, and OP72_reverse_R9Hmut 5′-GGACAGTCCAGAAGATGCACAGG. Sequencing data were analyzed using SnapGene software.

### *In vivo* studies

*In vivo* studies were carried out in compliance with the applicable laws, regulations, and guidelines at Labcorp Drug Development (Ann Arbor, MI, USA) or Epistem Ltd. (Manchester, UK). For each study, mice were checked regularly for health status and body weight and were sacrificed when predetermined termination criteria were reached.

#### NALM6

Groups of (*n* = 5) 6–8-week-old female NSG (NOD.Cg.Prkdc^scid^IL2rg^tmWjl^/SzJ− Jackson Laboratory, Bar Harbor, ME, USA) mice were injected intravenously (i.v.) on day 3 with 5 × 10^5^ NALM6 B-cell acute lymphoblastic leukemia cells expressing Luciferase (NALM6-Luc-mCh-Puro) (ATCC/Labcorp, Burlington, NC, USA). Three days after tumor injection, mice were injected with luciferin intraperitoneally (i.p.) and imaged under anesthesia using an IVIS S5 Imaging System, with bioluminescence imaging (BLI) data analyzed using Living Image 4.7.1 software (both from Perkin Elmer, Waltham, MA). Using these BLI measurements, mice were distributed into groups, ensuring that the mean tumor burden for each group was within 10% of the mean tumor burden for the study population. The mice were untreated or injected i.v. with 1 × 10^6^, 5 × 10^6^, or 2 × 10^7^ human T cells transduced with a vector to express 7G5.TCR after knocking out the endogenous TCR. IVIS imaging was then performed 3 days later, and twice each week thereafter, to follow tumor progression. Imaging data were obtained within 10 min after luciferin injection.

#### A375-MR1

A375 melanoma cells (ATCC) were transduced with lentiviral particles encoding B2m-MR1*01 to express high levels of MR1*01 at the cell surface. NSG mice were subcutaneously (s.c.) injected with 5 × 10^6^ A375-MR1 cells. The mice were randomized 1 day later into groups of eight animals and injected i.v. with vehicle, 2.3 × 10^7^ nontransduced (NTD), or either 2.3 × 10^7^ or 1.2 × 10^7^ T cells from two different donors (equivalent to 1 × 10^7^ or 5 × 10^6^ T cells expressing 7G5.TCR-T). For this experiment, the endogenous TCR was not knocked out. Mice were weighed and tumor volume was assessed using calipers three times weekly.

## Results

### The TCR from MC.7.G5 in TCR-T format effectively recognizes multiple cancer cell lines

**Figure 1:**
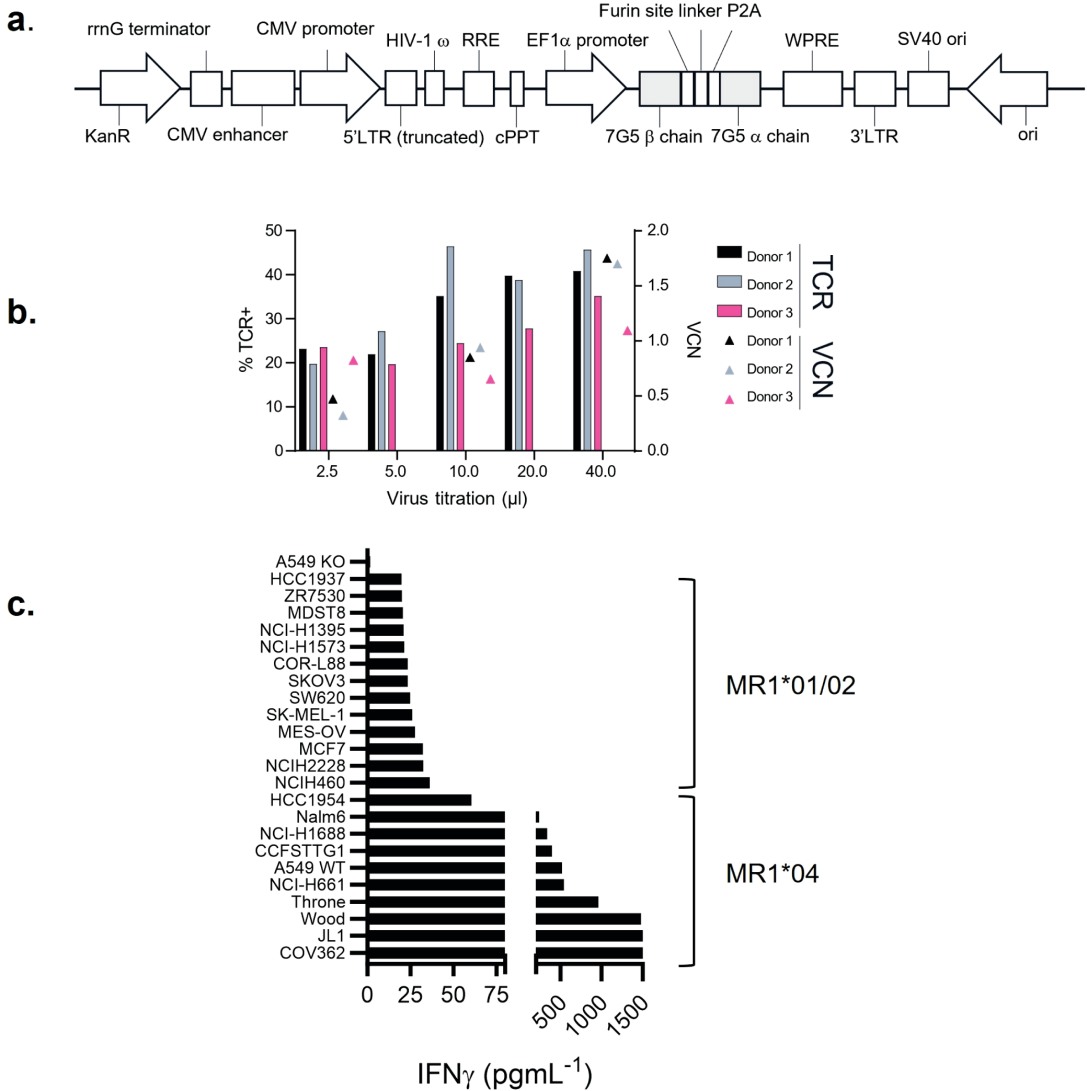
7G5.TCR-T preferentially targets cancer cells bearing allelic variant MR1*04. **(A)** Features of the lentiviral vector (LVV) used to transduce the 7G5.TCR into primary T cells. The 7G5.TCR used murine constant regions to enhance chain pairing and cell surface expression in primary T cells. A detailed map is shown in **Supplementary Figure S1**. **(B)** Transduction efficiency of 7G5-encoding LVV in healthy CD8 T cells derived from PBMC. Staining of 7G5.TCR-T for TCRBV25 was used to assess 7G5 expression. *x*-Axis indicates the volume of LVV supernatant used per transduction. The right *y*-axis indicates the viral copy number (VCN). **(C)** Reactivity of 7G5.TCR-T to 24 cancer cell lines (of 133 analyzed, **Supplementary Table S1**) ranked as measured by IFN-γ ELISA at 48 h following co-culture of 20,000 target cells and 60,000 7G5-TCR-T; E:T ratio: 3:1.

A T-cell clone, MC.7.G5, has been described as MR1-restricted, exhibiting pan-cancer reactivity, and failing to recognize normal cells or cells subject to various forms of cellular stress (25). To test the potential of the MC.7.G5 TCR for cancer therapeutic translation, the MC.7.G5 TCR was cloned into a lentiviral vector for transduction into human T cells (**Figure 1A**; **Supplementary Figure S1**). Transduction of the native TCR sequence derived from MC.7.G5 (“nat.7G5”) resulted in poor cell surface expression in primary human T cells (expressing endogenous TCRs) (data not shown). Previous studies have shown that murinization of TCR constant domains and substitution of hydrophobic residues near the transmembrane domain enhance cell surface expression while maintaining TCR specificity (31). To test if this strategy would enhance cell surface expression of the MC.7.G5-derived TCR on T cells, we murinized the constant domains of the vectored TCR sequence and introduced hydrophobic residues into the transmembrane portion of the alpha chain. The locations of the hydrophobic residues are depicted in **Supplementary Figure S1A**. This resulted in enhanced expression of the murinized MC.7.G5-derived TCR (“7G5”) at the cell surface (**Supplementary Figure S1B**) and was consistently detected in 20%–50% of healthy donor CD8+ T cells (**Figure 1B**).

### 7G5.TCR-T does not demonstrate pan-cancer reactivity

**Figure 2:**
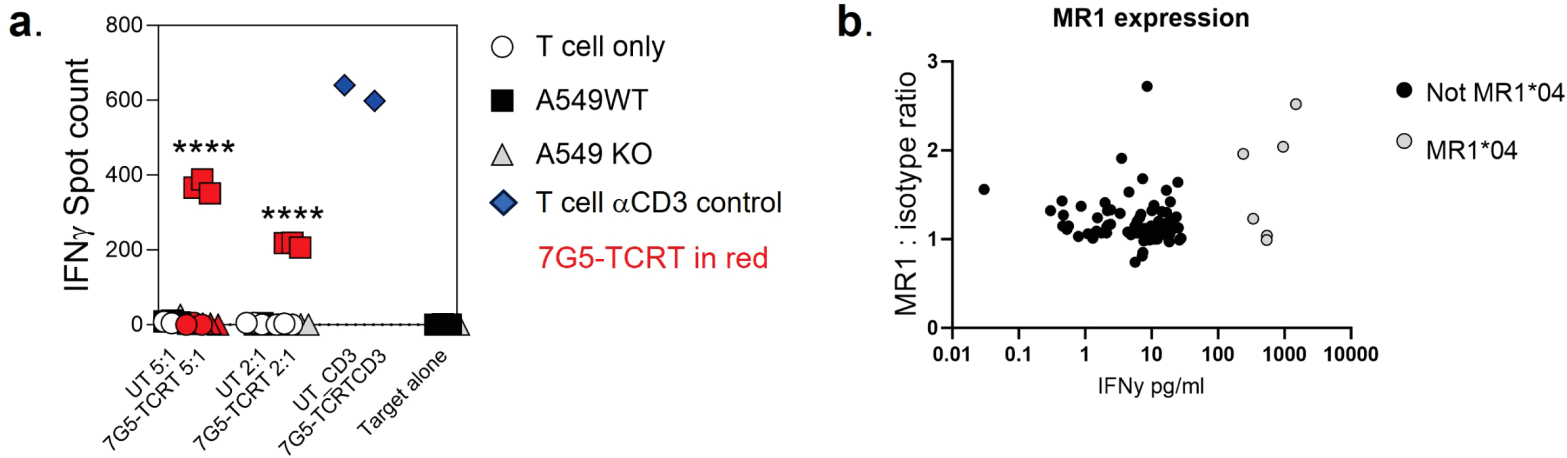
**(A)** Potency of 7G5.TCR-T against MR1-expressing NSCLC line A549 or A549 MR1 knockout cells. ELISpot assays were used to measure IFN-γ secretion by 7G5.TCR-T cells in response to the targets at the indicated effector-to-target ratios. Representative IFN-γ ELISpot showing reactivity of 7G5.TCR-T to MR1 wild-type or MR1 knockout A549 cells. The data shown in **(A)** is representative of at least six biological replicates. ^****^ *p* < 0.0001 statistical difference between reactivity seen with 7G5.TCR-T against A549WT cells versus A549.MR1KO, by one-way ANOVA with Tukey’s multiple comparison test. **(B)** MR1 expression on cancer cell lines as compared to isotype control, plotted against 7G5.TCR-T reactivity data from one donor (IFNy data expression is normalized to a response from nontransduced T cells). Data are shown as IFN-γ production from T cells following co-culture with cancer cell lines. Each point is an individual cancer cell line. Grey circles are MR1*04 cell lines, and black circles are non-MR1*04. There is no significant correlation between IFN-γ production and MR1 expression datasets as computed using nonparametric Spearman’s correlation and a two-tailed *t*-test (*p* = 0.58).

We next tested the hypothesis that the 7G5 TCR has pan-cancer recognition ability, in line with the reported activity of the parent CD8^+^ clone MC.7.G5 (25), by measuring the reactivity of 7G5.TCR-T to an extensive panel of cancer cell lines. We set up reactivity assays of 7G5.TCR-T on 133 cancer cell lines, using IFN-γ as a readout of activity. We observed convincing 7G5.TCR-T reactivity, defined as > 50 pg/ml IFN-γ, in co-culture with only 7% (nine of 133) of cancer cell lines (**Figure 1C**; **Supplementary Table S1**). We tested several hypotheses to establish the attributes required for cancer cells to be recognized by 7G5. Although reactivity strictly required expression of MR1 (**Figure 2A**), reactivity of 7G5.TCR-T did not correlate with surface levels of MR1 on cell lines tested (**Figure 2B**), the number or polarization of mitochondria, levels of superoxide within the cell, cell cycle time, or transcriptional levels of the alternate MR1 transcripts A and B (data not shown). Based on a recent report of multiple polymorphic forms of MR1, we investigated whether specific allomorphs of MR1 were recognized preferentially by 7G5.TCR-T (9).

### The 7G5 T-cell receptor preferentially targets cancer cells bearing the MR1*04 allele

To investigate whether 7G5.TCR-T is preferentially restricted by previously described MR1 variants (9, 12), we PCR-amplified and sequenced the MR1 gene from the 133 lines tested (**Supplementary Table S1**). 7G5.TCR-T reacted comparatively poorly to lines A375 and NCI-H1755, which express MR1*01 but not MR1*04 (**Figure 1C**). Using IFN-γ as a measure of reactivity of 7G5.TCR-T to these cancer lines, we showed definitively that 7G5.TCR-T preferentially targets cells expressing the MR1*04 allele, while responses to over 100 MR1*01- and MR1*02-expressing cells were typically much weaker or nonexistent. IFN-γ production by 7G5.TCR-T to MR1*04-bearing targets was between two- and 110-fold greater than to those not expressing this allele (**Figure 1C**; **Supplementary Table S1**). The lack of activity of 7G5.TCR-T against MR1*02-expressing cells shows that the H17R mutation is not sufficient for recognition of MR1*04.

### 7G5.TCR-T only significantly responds to MR1*01 on target cells at supraphysiological expression levels

**Figure 3:**
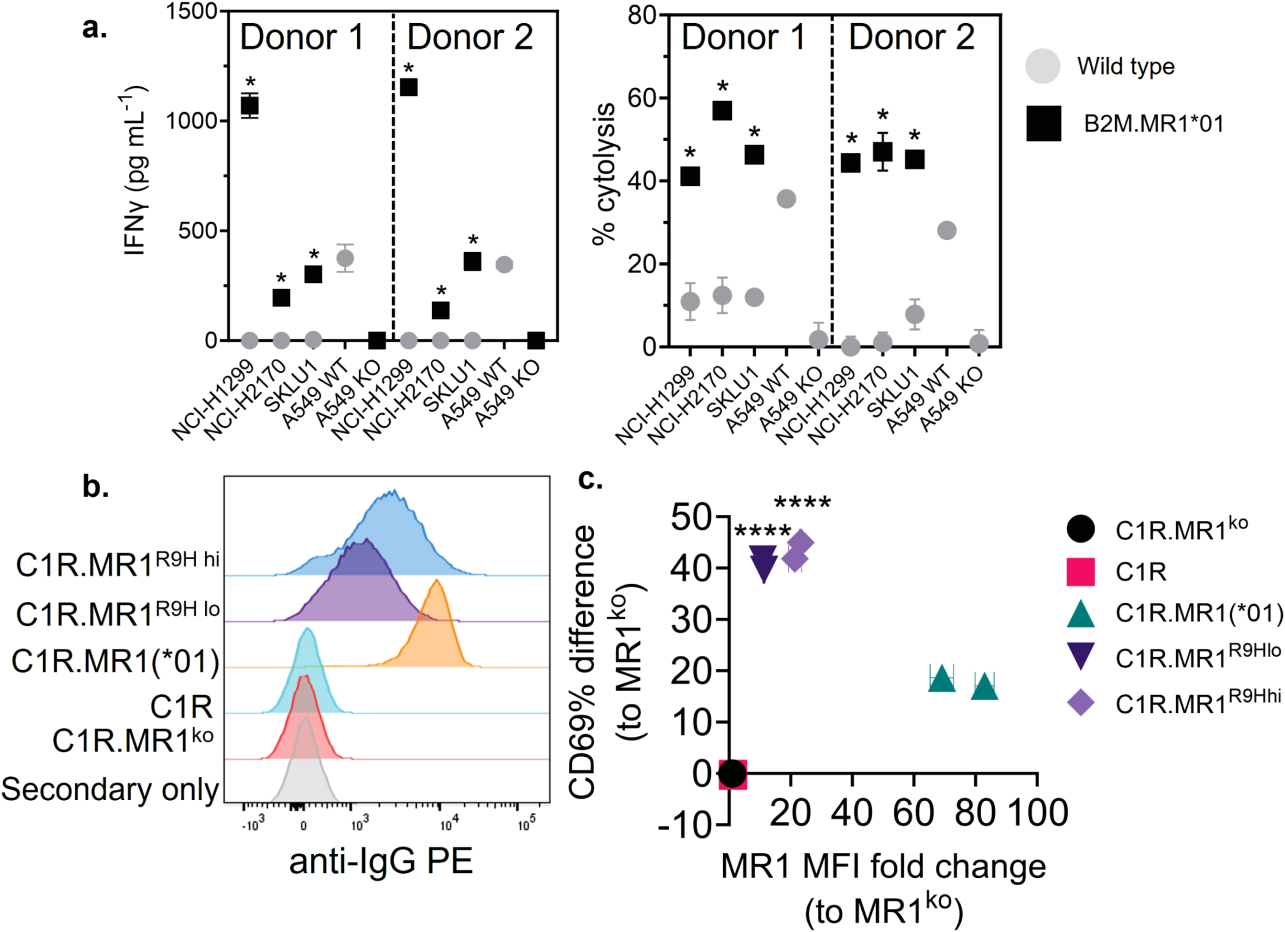
Overexpression of MR1*01 is required for reactivity of 7G5.TCR-T to cells lacking MR1*04. **(A)** Left panel: Mean (± SD, *n* = 3) IFN-γ concentration measured by ELISA in the supernatant 18–24 h following co-culture of 20,000 WT or β2m. MR1-expressing cancer lines (*x*-axis) and 100,000 7G5-TCR-T (E:T ratio: 5:1). MR1 haplotypes of the cells investigated; NCI-H1299 and NCI-H2170: MR1*01; SKLU1: MR1*02; A549: MR1*04/*01 heterozygous. Right: Mean (± SD, *n* = 3) percentage cytolysis above the background of WT or β2m. MR1-expressing cancer cell lines (*x*-axis) by 7G5.TCR-T cells from two donors after 24 h of co-culture. Cell numbers varied depending on target but effector-to-target ratios (E:T) remained at 5:1. ^*^ *p* < 0.05—significant difference between the B2M.MR1*01 and wild-type groups, as assessed by a two-tailed Wilcoxon matched-pairs signed rank test. **(B)** MR1 expression of C1R derivatives. Cells were stained with 8F2.F9 hybridoma supernatant, followed by antimouse IgG-PE. **(C)** Activation of Jurkat.β2m^ko^.7G5 measured as %CD69^hi^ (after subtraction of the mean %CD69^hi^ on stimulation with C1R.MR1^ko^ cells) vs. fold change of the median fluorescence intensity of MR1 expressed by the stimulating APC compared to C1R.MR1^ko^. Data are from two independent experiments. Each point represents the mean (± SD) of three in-experiment replicates for CD69 and two in-experiment replicates for MR1. ^****^Both C1R.MR1^R9Hlo^ and C1R.MR1^R9Hhi^ significantly different from C1RMR1 (^*^01) *p* < 0.0001 assessed by two-way ANOVA with Tukey’s multiple comparisons test.

We next explored the possibility that the preferential reactivity of 7G5.TCR-T to MR1*04 allele-expressing cancer cells might be due to MR1*04 being more abundantly expressed at the cell surface than MR1*01. As we did not observe any correlation between surface expression of MR1 allomorphs on cancer cell lines, as measured by flow cytometry, and 7G5.TCR-T efficacy, as measured by IFN-γ release (**Figure 2B**), we concluded that MR1*04 expression stimulates 7G5.TCR-T to a much greater extent than MR1*01 when the levels of expression on the cell surface are comparable. Given most studies to date with MR1-reactive T cells rely on MR1*01 overexpressing lines as targets, we next investigated whether 7G5.TCR-T could react to MR1*04-negative cancer lines engineered to express supraphysiological levels of the MR1*01 allele. The cancer cell lines NCI-H1299, NCI-H2170, and SKLU1, all negative for MR1*04 expression, were chosen as they are poor targets for 7G5.TCR-T (**Supplementary Table S1**). Overexpression of MR1*01 in these cells resulted in 7G5.TCR-T-producing IFN-γ and killing the cells at a level equivalent to the reactivity seen with A549, which is heterozygous for MR1*01/MR1*04 (**Figure 3A**). To confirm the *in vitro* findings and mitigate against cell culture artifacts impacting *in vitro* results, two different *in vivo* models were established in immune-compromised mice. One utilized NALM-6-luciferase, an aggressing leukemic model naturally heterozygous for MR1*04. The second model utilized the melanoma xenograft A375 (MR1*01 homozygous) engineered to express supraphysiological levels of MR1*01. Both models demonstrated a robust delay in tumor progression compared to control untransduced donor T cells (**Supplementary Figure S2**). Although activated by supraphysiological levels of MR1*01, we investigated if the R9H substitution that differentiates MR1*01/MR1*02 and MR1*04 could further increase 7G5 activation. Previously generated C1R overexpressing MR1^R9H^ (C1R.MR1^R9H^) (12), were sorted for low and high expression (C1R.MR1^R9Hlo^ and C1R.MR1^R9Hhi^) and used to activate Jurkat.β2m^ko^ expressing the 7G5 TCR (Jurkat.β2m^ko^.7G5 cells). Despite overall lower MR1 surface expression than C1R.MR1-, C1R.MR1^R9Hlo^-, and C1R.MR1^R9Hhi^-activated Jurkat.β2m^ko^.7G5 to a greater extent than C1R.MR1, as measured by CD69 surface expression (**Figures 3B, C**; **Supplementary Figure S3**). Although MR1*04 expression appears to drive recognition by 7G5, the expression of MR1*04 is not the only component driving recognition; T-cell reactivity varies between cell lines and is not directly linked to MR1 allomorph levels. Also, the levels of costimulatory and inhibitory molecules at the surface of the tumor cell and the expression levels of β2M will affect the reactivity. Therefore, it is likely that the ligand presented by MR1*04 also plays a role in the differences seen in T-cell recognition of target lines. This is exemplified by the lower levels of IFN-γ secretion in response to the MR1*04 cancer line HCC1954.

### 7G5 reactivity is similar to other MR1-restricted TCRs for allele and ligand discrimination

**Figure 4:**
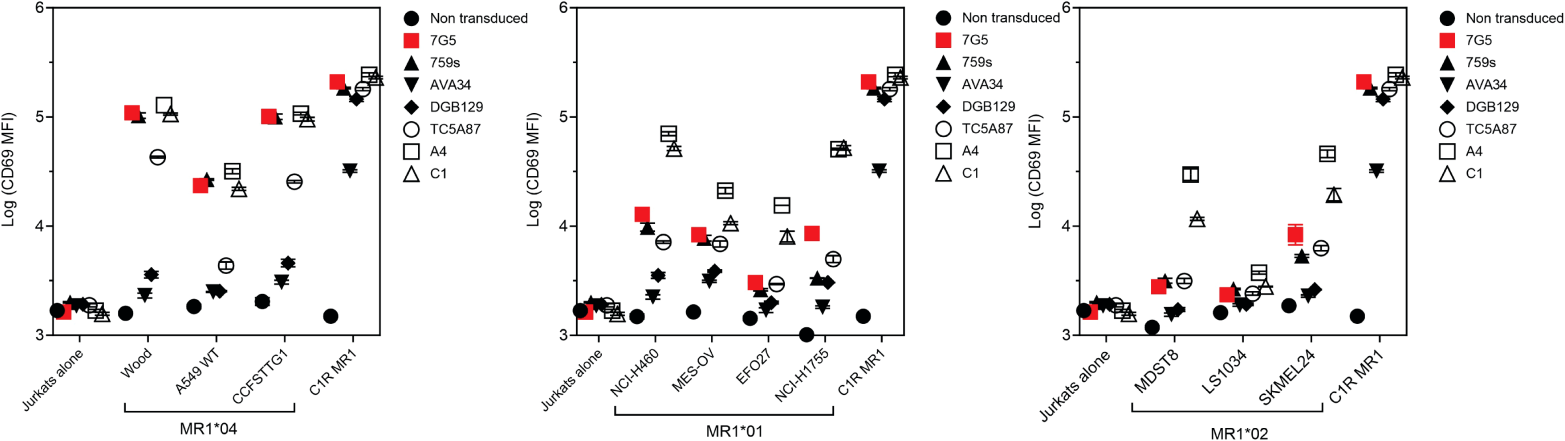
Comparison of MR1 allele restriction and ligand reactivity of 7G5.TCR-T with other MR1 reactive TCRs. Log median fluorescence intensity values of CD69 surface levels on Jurkat cells expressing one of seven T-cell receptors (*y*-axes) after 24-h incubation with MR1*01-, MR1*02-, or MR1*04-expressing cancer cell lines (*x*-axes). Jurkat cells were incubated alone or with C1R cells overexpressing MR1*01 as negative and positive controls, respectively. Geometric mean (*n* = 3) values are plotted. Data are representative of two experimental repeats.

MR1-restricted T cells show diverse TCR usage and exhibit varying distribution, cell reactivity, and ligand preferences. We investigated whether the preferential MR1*04 reactivity seen with the 7G5.TCR-T was shared by TCRs derived from additional T-cell clones (759S, A4, and C1; called “7G5-like” here). The 759S TCR was derived from the T-cell clone MC.27.759S as previously described (25), and TCRs A4 and C1 were derived from T-cell clones isolated by a similar methodology to the identification of the MC.27.759S clone. We also compared the reactivity of 7G5.TCR-T with MR1-reactive TCRs derived from T-cell clones AVA34, DGB129, and TCA5A87, reported elsewhere (32), raised with different methodology, and termed “MR1T”. Jurkat cells CRISPR-engineered for deletion of endogenous TCR and β2M (to prevent aberrant TCR chain pairing and recognition of MR1 *in trans* on adjacent Jurkat cells) were transduced with the described TCRs and co-cultured with cancer cell lines expressing MR1*01, MR1*02, MR1*04, or C1R cells engineered to express supraphysiological MR1*01. Jurkat reactivity, as measured by CD69 upregulation, fell into three main categories (**Figure 4**; **Supplementary Figure S4A**). Jurkat cells expressing 7G5, the 7G5-like TCR 759S, or the MR1T TCR TC5A87 displayed a similar pattern of reactivity against cancer cell lines, exhibiting elevated reactivity to MR1*04 cells and limited reactivity to MR1*01 and MR1*02 lines. Jurkat cells expressing one of the remaining 7G5-like TCRs, A4 or C1, reacted to MR1*04 but also showed greater CD69 upregulation to some cell lines expressing MR1*01 and MR1*02, compared to 7G5. Jurkat cells expressing the MR1T cell-derived TCRs AVA34 or DGB129 showed no apparent reactivity to cancer cell lines with physiologic MR1 expression. All the TCR-engineered Jurkat cells were robustly reactive to the positive control cells, C1R, expressing supraphysiological levels of MR1*01.

Collectively, these data point to the 7G5-like TCRs and possibly TC5A87 having activity restricted to MR1*04-expressing cell lines in the endogenous MR1 setting. Some putative metabolite ligands specifically recognized by MR1T have been reported (27). We asked whether 7G5-like TCRs could recognize these ligands (3*Z*,5*E*)-6-((9*H*-purin-6-yl) amino) hexa-1,3,5-triene-1,1,3-tricarbaldehyde (“M3ADE”) and 6-((2*R*,4*S*,5*R*)-4-hydroxy-5-(hydroxymethyl)tetrahydrofuran-2-yl)-3-(2-oxoheptyl)-1,8a-dihydroimidazo[1,2-*c*]pyrimidin-5(6*H*)-one (“ONEdC”) presented on C1R cells, which naturally express MR1*01. Using the same Jurkat system, we showed that the TCRs derived from MR1T lines DGB129 and AVA34 recognized C1R targets incubated with M3ADE, as reported previously (33). Preincubation of C1R cells with M3ADE or ONEdC did not render C1R cells sensitive to the remaining TCRs, suggesting these ligands are not responsible for 7G5.TCR-T, 7G5-like TCRs, or the MR1T TC5A87 liganded MR1 recognition (**Supplementary Figures S4B**, **S5**).

### 7G5.TCR-T function is inhibited by the bacterial ligand acetyl-6-formylpterin

**Figure 5:**
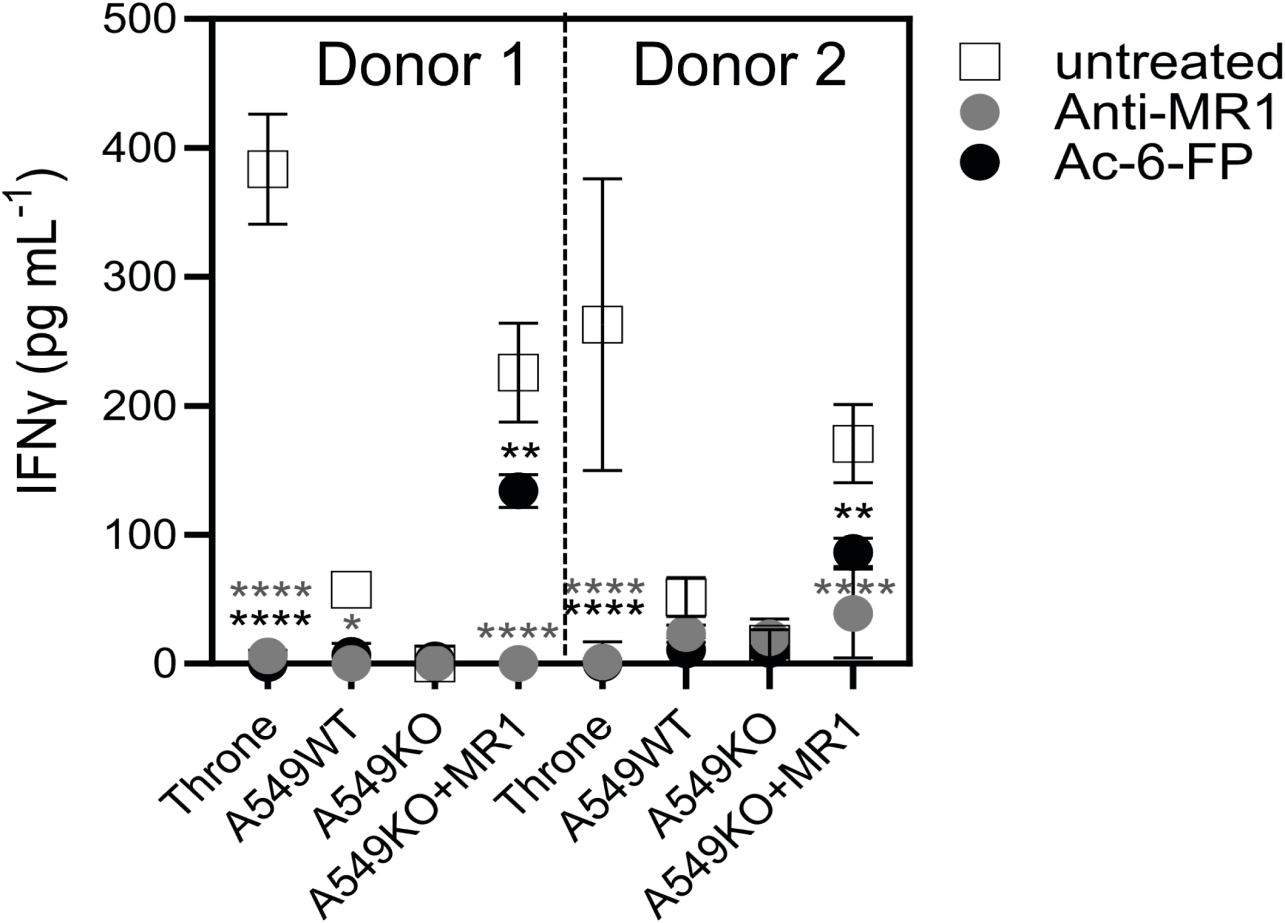
7G5.TCR-T function is inhibited by the bacterial ligand 6-acetyl-formylpterin. Reactivity of 7G5.TCR-T derived from two donors to cancer lines expressing MR1*04, or A549-MR1KO-negative control cells in the presence or absence of Ac-6-FP or blocking anti-MR1 antibody IFN-γ were measured by ELISA at 18 h following co-culture of 20,000 target cells and 60,000 7G5-TCR-T (E:T ratio: 3:1). Two-way ANOVA was performed with Dunnett’s multiple comparisons *post-hoc* test. Statistical significance compared to the untreated uncontrol is indicated as ^*^ *p* < 0.05, ^**^ *p* < 0.01, or ^****^ *p* < 0.0001.

As the 7G5 TCR, in the context of the MC.7.G5 clone, appears to depend on the key lysine residue present in the ligand-binding groove at position 43 of the mature MR1 protein (K43) for its activation (25) it is likely that this recognition is ligand-dependent. The lysine at position 43 forms a Schiff base with certain metabolites, anchoring them in the MR1-binding cleft (1, 2, 14). K43 resides deep in the A’ pocket of the MR1-binding groove and is unlikely to be easily accessible to TCRs. We sought to exclude the possibility that the 7G5 TCR recognizes MR1*04 in a ligand-agnostic or ligand-independent fashion. We reasoned that if 7G5 binds to MR1*04 in a ligand-agnostic fashion, then saturating the surface MR1 of cancer lines expressing MR1*04 with the bacterial ligand acetyl-6-formylpterin (Ac-6-FP) should not block recognition. Ac-6-FP was previously shown to block the activity of the MC.7.G5 T-cell clone (25). Howson et al. demonstrated that Ac-6-FP is presented in the context of MR1 with an R9H substitution; however, the MAIT-stimulating ligand 5-OP-RU was not presented by MR1 R9H (12). For MR1*04 cell lines (Throne and A549), Ac-6-FP abrogated the response of 7G5.TCR-T as measured by IFN-γ production (**Figure 5**). Thus, these data would support the idea that the 7G5 TCR in the context of TCR-T does not recognize MR1 when the ligand-binding pocket is occupied with Ac-6-FP. As both Throne and A549 are MR1*01/*04 heterozygotes, it is not possible to discriminate between recognition of these allomorphs in this experiment. However, binding was totally abrogated in both cell types by the addition of Ac-6-FP.

### T cells transduced with 7G5, and 7G5-like T-cell receptors are activated by MR1*04 expressing healthy noncancer cells

**Figure 6:**
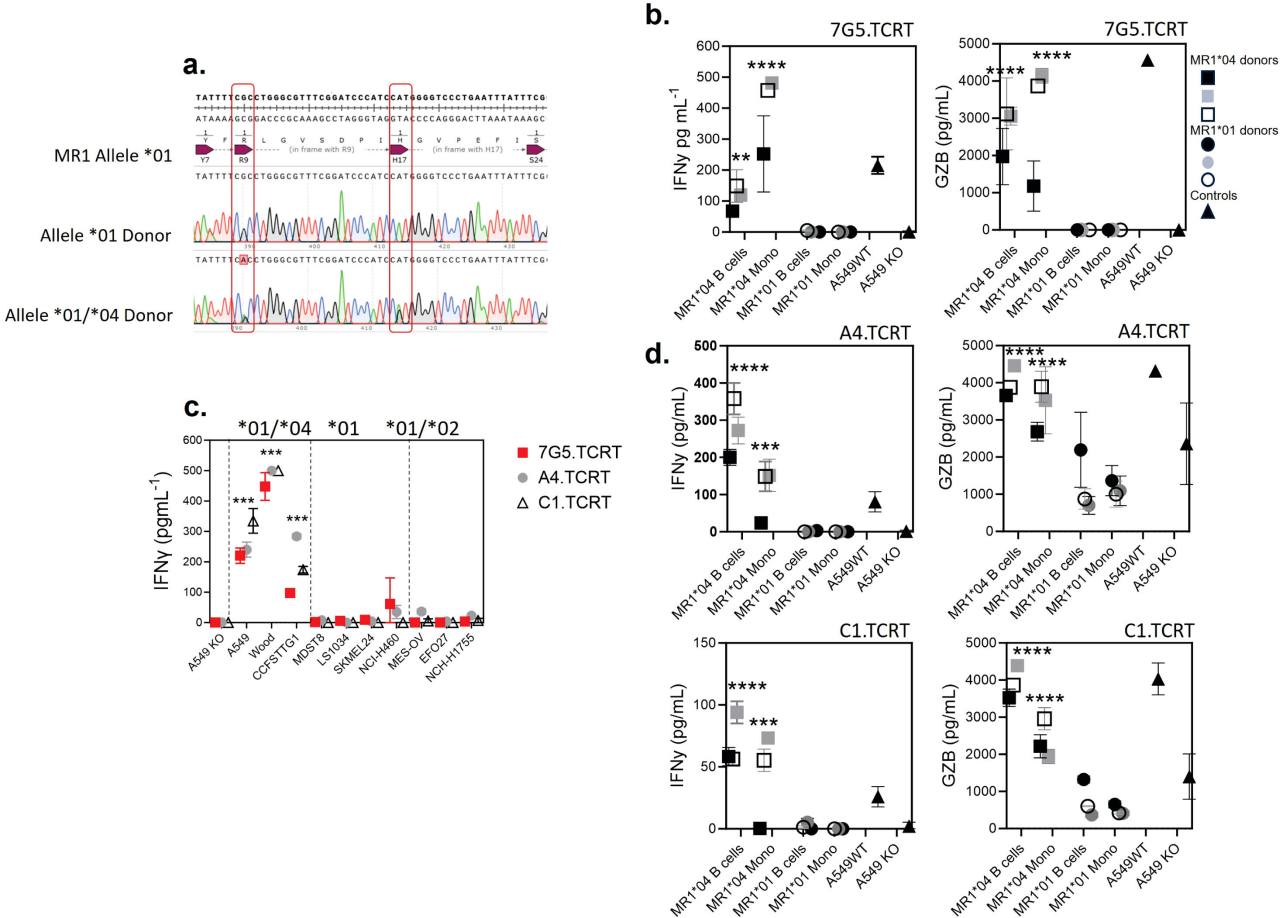
T cells transduced with 7G5 and 7G5-like TCRs are activated by MR1*04 expressing healthy cells. **(A)** Sanger sequencing of the MR1 locus of two of 200 blood donors analyzed. The MR1*01 allele and MR1*04 allele differ by two nucleotide substitutions leading to R9H/H17R substitutions in the MR1*04 allele. The lower row shows a donor heterozygous for MR1*01/*04, note the miscalling of the mixed nucleotides at both positions. **(B)** Reactivity of one representative donor of three 7G5.TCR-T donors to monocytes and B cells derived from PBMC from three MR1*01/*04 heterozygous blood donors, and three MR1*01 homozygous donors. IFN-γ (left) and granzyme B (right) were measured by ELISA at 48 h following the co-culture of 40,000 PBMC-derived cells and 200,000 7G5.TCR-T (E:T ratio: 5:1). Grey, black, and white squares represent monocytes and B cells from three MR1*01/*04 donors. Grey, black, and white circles represent monocytes and B cells from three MR1*01 donors. Black triangles represent the positive and negative controls A549 and A549.MR1KO, respectively, one donor with nine technical replicates. ^**^ *p* < 0.01 and ^****^ *p* < 0.0001—significant differences between reactivity to MR1*04 targets vs. MR1*01. **(C)** Reactivity of the 7G5.TCR-T (red) and TCR-T expressing the 7G5-like TCRs A4 and C1 to cancer cell lines homozygous for MR1*01 or heterozygous for MR1*01 and MR1*02 or MR1*04. Reactivity was measured with IFN-γ ELISA at 48 h following the co-culture of 20,000 cancer cells and 60,000 7G5.TCR-T (E:T ratio: 3:1), and data shown are representative of two biological repeats with two separate donors for TCR-T. ^***^ *p* < 0.001 by one-way ANOVA with Tukey’s multiple comparison test indicating significant differences between 7G5 and 7G5-like TCR reactivity to MR1*04 expressing targets compared to MR1*04-negative cells. **(D)** A4-TCR-T and C1-TCR-T reactivity of one representative of three TCR-T donors to monocytes and B cells derived from PBMC from three MR1*01/*04 heterozygous blood donors and three MR1*01 homozygous donors. IFN-γ and granzyme B were measured by ELISA at 48 h following the co-culture of 40,000 PBMC-derived cells and 200,000 7G5-TCR-T (E:T ratio: 5:1). Grey, black, and white squares represent monocytes and B cells from three MR1*01/*04 donors. Grey, black, and white circles represent monocytes and B cells from three MR1*01 donors. Black triangles represent the positive and negative controls A549 and A549.MR1KO, respectively, from one donor with nine technical replicates. ^***^ *p* < 0.001 and ^****^ *p* < 0.0001—significant difference between reactivity to MR1*04 targets vs. MR1*01.

Given that the allele frequency of MR1*04 heterozygotes is estimated to be approximately one in 100 in European Caucasians (9), we reasoned that previous characterization of the MC.7.G5 T-cell clone’s inability to be activated by healthy cells would likely have been performed on cells derived from MR1*01 or *02 allele-expressing donors (25). We next tested two alternative hypotheses: either 7G5.TCR-T recognizes a cancer-specific/enriched ligand restricted by MR1*04 or binds preferentially to a ligand presented by all cells expressing the MR1*04 allele. We sequenced the MR1 locus of ~ 200 healthy blood research donors for MR1 allele identities. We identified four donors who were heterozygous for MR1*01/*04 (example plots in **Figure 6A**). To conduct reactivity assays of 7G5.TCR-T against healthy, benign blood cells, we isolated monocytes and B cells from three MR1*01/*04 donors as cell types that express the highest cell surface levels of MR1 in blood. 7G5.TCR-T did not produce IFN-γ or granzyme B in response to monocytes and B cells from MR1*01 homozygous donors. In contrast, significant amounts of both IFN-γ and granzyme B were produced in co-cultures with MR1*01/*04 heterozygous donor-derived B cells and monocytes, suggesting that 7G5 TCR-T recognizes a ligand(s) that is present both in tumor cells and normal cells expressing MR1*04 (**Figure 6B**), similar to that observed in Lepore et al. (24). Finally, we tested whether the 7G5-like TCR-T (A4.TCR-T and C1.TCR-T) had similar cancer and normal cell reactivity to 7G5.TCR-T. The three TCR-T react to cancer cells heterozygous for MR1*04 and only marginally or not at all to cells that express only MR1*01 or are heterozygous for MR1*01/*02 (**Figure 6C**). Again, this reactivity was not cancer-specific, with A4.TCR-T and C1.TCR-T producing IFN-γ and granzyme B in response to B cells and monocytes from MR1*04 heterozygous donors and not in response to MR1*01/02 donors (**Figure 6D**). When expressed in Jurkat cells, the A4 and C1 TCRs responded to MR1*01-expressing tumor cell lines (**Figure 4**), as shown by upregulation of CD69. This apparent discrepancy is likely due to the TCR signal strength required to trigger the upregulation of CD69 in Jurkat cells anticipated to be lower than that required to trigger IFN-γ production by primary T cells.

## Discussion

In recent years, MR1 has held great promise as a target for TCR-mediated tumor immunotherapy. This is largely because it was considered to be monomorphic and invariant across all populations. MR1-restricted T-cell clones have also been identified, which could kill multiple cancer lines without recognizing healthy, noncancer cells, therefore raising the potential of translating TCR-based, MR1-restricted therapy for cancer patients (24–26). The discovery of rare T-cell clones capable of recognizing potential “pan-cancer” MR1-restricted ligands has led to intense efforts to learn more about the biology of MR1 and MR1-restricted T cells in cancer. Recently, it has been shown that MR1 has at least six alleles. The substitution R9H associated with the MR1*04 allele potentially skews the ligands that are able to be presented by the MR1*04 protein, as inferred by the observation that it is unable to present 5-OP-RU (9, 12). We set out to test the idea that therapeutic TCR-T cells could be generated, expressing TCRs from T-cell clones able to kill cancer cells from multiple tumor types while leaving benign cells untouched (24, 25).

In this study, we have shown that the 7G5.TCR derived from the MC.7.G5 T-cell clone and other MR1-restricted TCRs with similar properties (e.g., tumor cell recognition and MR1 K43-dependence) robustly redirect T cells to kill cancer cells in the TCR-T format both *in vitro* and *in vivo*. The 7G5.TCR-T induces tumor regression in a solid tumor model of melanoma in NSG mice and also shows clear survival benefits and substantial delay in tumor growth in a disseminated leukemic model in NSG mice, which added support to the *in vitro* observations in a complex model that lacked constituents of cell culture media. The improved efficacy of 7G5.TCR-T in the solid tumor model compared with the disseminated leukemic model was likely due to the differences in aggressiveness of *in vivo* growth between the cell lines but also to the fact that the melanoma model was overexpressing MR1, leading to likely supraphysiological levels of cell surface expression. While validating the 7G5.TCR-T, we observed that robust activity was not pan-cancer in nature, being restricted to a minority of cancer cell lines. Efforts to identify a biomarker for reactivity of 7G5.TCR-T led to the discovery that one rare (approximately 1:100) allele of MR1, MR1*04, predicted cancer cell line reactivity. Ultimately, this led to the determination that 7G5.TCR-T was not cancer-specific but reactive to normal B cells and monocytes heterozygous for MR1*04.

To our knowledge, this is the first demonstration of an MR1-restricted TCR that has MR1 allomorph specificity. Although the ligands for 7G5.TCR-T and related TCRs have not yet been identified, we demonstrated that the ligand is likely required to be bound to the MR1-binding groove for 7G5.TCR-T reactivity. We have also shown that 7G5.TCR-T TCRs do not recognize previously described MR1T ligands at physiological levels of MR1*01 expression in cancer cell lines. A recent study (33) demonstrated that conventional MAIT cells from healthy donors have the propensity to be promiscuous in their recognition. Such cells express markers of conventional MAIT cells and become activated in response to multiple ligands presented by MR1*01 overexpressed on cancer lines. These cells can react to healthy monocyte-derived dendritic cells, but the ligands recognized in this case remain to be identified.

The MR1*04 allele differs from MR1*01 by two nucleotide variants, resulting in an R9H substitution in the ligand-binding cleft and an H17R substitution in the α-1 domain outside the binding cleft. Howson et al. identified a homozygous MR1 mutation MR1^R9H/R9H^ in a patient with a primary immunodeficiency that was characterized by tattoo-associated human papillomavirus-positive (HPV+) warts (12). The patient displayed selective loss of all MR1-restricted MAIT cells due to this mutation, which caused structural changes to the ligand-binding pocket of MR1. This mutation accommodates the binding of Ac-6-FP but precludes binding of the stimulatory riboflavin-based MAIT ligand 5-(2-oxopropylideneamino)-6-d-ribitylaminouracil (5-OP-RU) and the subsequent ability of antigen-presenting cells to activate in an MR1-restricted manner. Lack of binding to 5-OP-RU results in a loss of ability to upregulate MR1^R9H^ in response to this ligand. The authors showed the patient had an expanded Vγ9/Vδ2+T-cell population, which may have arisen to compensate for the loss of circulating MAIT cells. The R9 residue of MR1 is a known MAIT TCR contact, as shown in the crystal structure data of MR1 binding the drug diclofenac (21) and as such may be involved in binding to other ligands. Our studies show that 7G5.TCR-T and 7G5-like TCRs can react to targets expressing supraphysiological MR1*01 and MR1*04, suggesting that either multiple ligands can be recognized by these TCRs or the same ligand is recognized but is presented preferentially with the histidine at position 9 in the binding cleft of MR1*04. The significance of MR1*02 for MR1T and MAIT cell function is unknown. MR1*02 does not appear to be recognized at physiological levels by 7G5.TCR-T. Approximately 25% of the population carries the MR1*02 allele, which incorporates the H17R substitution (9). The location of arginine 17, as revealed by the MR1 crystal structure (1), is distant to the binding cleft, so it is unlikely to be involved in antigen binding or TCR binding. It is possible that arginine 17 plays a role in MR1 trafficking or antigen loading, which might have applied evolutionary selection pressure to retain this allele. An alternative possibility is that this allele originally arose in a small population as a mutation with a founder effect and, upon mixing in larger populations, has remained without either selection or loss by drift.

The 7G5 TCR appears to be exquisitely sensitive to MR1*04 when the TCR is expressed either in Jurkat cells or primary T cells. The reactivity of the TCR when expressed on Jurkat cells is more sensitive than when expressed on primary T cells. This is likely due to the different readouts used in these experiments: CD69 expression on Jurkat vs. IFN-γ on primary T cells. A549 cells are one of the most stimulatory lines for 7G5.TCR-T, but MR1 (MR1*01 and/or MR1*04) is barely detectable on their surface by flow cytometry. This does not appear to be a technical artifact, as the antibodies most commonly used to detect MR1 by flow cytometry, 8F2.F9 (12) and 26.5, used in this study, both recognize MR1*01 and MR1*04 allomorphs (12). This reactivity contrasts with MR1T TCRs such as DGB129 (26) and TC5A87 (24), which in these studies appear to rely on targets that have been engineered to overexpress MR1 (26). This may simply reflect the methodology used to isolate such cells, with targets either overexpressing MR1 or not. Our data demonstrate that 7G5.TCR-T has up to 110-fold greater reactivity to MR1*04 but can still react to overexpressed MR1*01. As such, it is important for future studies to consider the potential biological implications of observations made with physiological vs. overexpressed MR1 protein.

We do not yet know the ligand(s) bound to MR1 required for 7G5.TCR-T activity. However, we do know that the putative MR1T ligands ONEdc or M3ADE do not activate 7G5.TCR-T or TCRs identified using comparable methods. This is perhaps unsurprising as previous work has shown that, unlike the MR1T cells, the 7G5 TCR relies on the MR1-binding groove residue K43 for ligand recognition (25), likely through a Schiff base formed with the ligand. For its role in tumor surveillance, our data challenge the current hypothesis that the ligands seen by MR1T and 7G5-like T cells are cancer-specific metabolites (25). Our findings suggest that the ligand(s) of 7G5 are common to normal (24) and transformed cells, challenging this recent dogma, or the TCR may be recognizing more than one ligand.

The original MC.7.G5 T-cell clone is heterozygous for MR1*01/*02 (**Supplementary Figure S6**) and therefore would not have been negatively selected for MR1*04 in the thymus. The patient with the reported MR1^R9H/R9H^ genotype had no detectable MAIT cells (12). This suggests that being homozygous for this polymorphism, and perhaps for MR1*04, may not be compatible with the thymic-positive selection of MR1-restricted T cells. It is clear from our data that healthy individuals lacking MR1*04, such as the donor of MC.7.G5, can harbor T cells that are not negatively selected in the thymus and can recognize a ligand or ligands derived from the normal metabolome or proteome and restricted by MR1*04. This finding has potential implications for transplantation. It is conceivable that allogeneic transplantation from donors lacking MR1*04 to those expressing this allele could pose a risk of GvHD. Conversely, transplantation of organs from MR1*04 donors to negative recipients may represent a higher risk of allogeneic rejection.

The promise of MR1-restricted TCR-based therapy for cancer has arisen through the discovery of T cells from human donors that appear to be restricted to MR1-ligand targets preferentially found in cancer. These data demonstrate that for TCRs derived from T-cell clones such as MC.7.G5, specificity appears to be restricted to a relatively rare MR1 allele bearing two SNVs and found in approximately 1% of the human population. This observation is important for three reasons. One, it highlights the need for a deeper understanding of non-MAIT, MR1-restricted TCRs prior to clinical translation; two, it raises the importance of deciphering the ligands driving reactivity in cancer and infection; and finally, it raises the specter that MR1 allele variants warrant understanding in the context of allotransplantation.

## Data Availability

The raw data supporting the conclusions of this article will be made available by the authors, without undue reservation.

## Supplementary material

Supplementary Table 1 Table showing 7G5.TCR-T reactivity as measured by IFN-γ ELISA to 133 cancer cell lines, including the MR1 allele expression of the lines. MR1 allele expression was derived from both public databases (CCLE and Crown Bioscience) and from internal PCR-sequences of DNA from these lines. Reactivity data from two separate T cell donors is shown. The table shows the top responded-to 10 lines followed by lines by indication type.

Supplementary Figure 1 7G5 expression cassette sequence. **(A)** Map of the 7G5 TCR expression cassette used in this study. Constant alpha and beta regions were murine and hydrophobic residues were introduced into the transmembrane region of the alpha chain as described in the text and denoted on the protein sequence. **(B)** Comparison of 7G5.TCR-T manufactured with murine or human TCR constant regions. Concentrated lentivirus was titrated using three T cell donors and 7G5 TCR expression, measured by TRBV25 expression (normal expression on only 1% of peripheral T cells).

Supplementary Figure 2 7G5.TCR-T effectiveness in murine tumor models. **(A)** NSG mice were implanted i.v. with NALM6-Luc tumor cells on day -3 and were then untreated (filled circles) or received 2x10^7^ non-transduced (NTD) T cells (filled squares) or different doses ranging from 2x10^7^-1x10^6^ of 7G5 TCR T cells (open symbols as indicated) from one healthy donor on day 0. Mice were injected i.p with luciferin imaged on day 0, day 3 and twice per week thereafter. In this study the T cells were transduced with a vector that expressed the 7G5 TCR and removed the endogenous TCR using CRISPR-Cas9. See methods section for details of CRISPR-Cas9 methodology. Data are shown as the mean +/-SD (*n* = 5 mice/group) of whole-body Bioluminescence Imaging (BLI) measurements (photons/second). Multiple student’s t test comparing all time points, untransduced versus 7G5 TCR with a dose of 2x10^7^ shows a significant difference, p<0.005 except at t=0. **(B)** NSG mice were injected subcutaneously (s.c.) with 5x10^6^ A375-MR1*01 cells. The following day, mice were randomised into groups and on the same day, were injected i.v. with vehicle (filled circles), or T cells from donor 1 or 2 (filled and open symbols). These were 2.3x10^7^ total NTD T cells (squares), or 1x10^7^ (triangles) or 5x10^6^ (diamonds) 7G5 TCR-transduced T cells. In this study the T cells were transduced with a vector that only expressed the 7G5 TCR and did not remove the endogenous TCR. Calliper tumour volume measurements were performed three times a week from Day 5. Data are shown as the mean tumour volume (mm3) +/- SD (n=8 mice/group). ANOVA test with Tukeys *post hoc* analysis shows a significant difference (p<0.001) between non-transduced T cells and ENA-0001 cells at all doses.

Supplementary Figure 3 Gating strategy for MR1 expression in C1R derivatives. Gating strategy for Jkt.β2m^ko^.7G5 activation assays. Gated for single, live, GFP^lo^, CD3^+^ Jkt.β2m^ko^.7G5 cells prior to assessment of CD69 expression. A) Shows example stimulation with C1R.MR1(*01) cells (GFP^hi^ APC). B) Shows example stimulation with C1R.MR1^R9Hlo^ cells (GFP^lo^ APC). C) Example scatter plots of Jkt.β2m^ko^.7G5 CD69 expression on stimulation with different APCs (left to right: No APC, C1R.MR1^ko^, C1R, C1R.MR1(*01), C1R.MR1^R9Hlo^, C1R.MR1^R9Hhi^).

Supplementary Figure 4 7G5 and 7G5-like TCR-T reactivity against cancer lines expressing MR1*01, MR1*02 and MR1*04 and MR1 ligands. **(A)** Heatmap showing log median fluorescence intensity values of CD69 surface levels on Jurkat cells expressing one of seven T cell receptors (Y axis) after 24-hour incubation with MR1*01, MR1*02 or MR1*04-expressing cancer cell lines (X axis). Jurkat cells were incubated alone or with C1R cells overexpressing MR1*01 as negative and positive controls respectively. Geometric mean (n=3) values are plotted. Data are representative of two experimental repeats. **(B)** Heatmap showing log median fluorescence intensity values of CD69 surface levels on Jurkat cells expressing one of seven T cell receptors (Y axis) after 24-hour incubation with C1R cells in the presence of the indicated ligand concentrations (X axis). Jurkat cells were incubated with C1R cells overexpressing MR1*01 as positive controls. Geometric mean (n=3) values are plotted. Data are representative of two experimental repeats.

Supplementary Figure 5 7G5-TCR-T and 7G5-like reactivity to MR1 ligands. Raw data from heat-map represented in **Supplementary Figure S4**. Graphical representation of heatmap data presented in **Supplementary Figure 4B** showing log median fluorescence intensity values of CD69 surface levels on Jurkat cells expressing one of seven T cell receptors (Y axis) after 24-hour incubation with C1R cells in the presence of the indicated ligand concentrations (X axis). C1R cells were incubated with C1R cells overexpressing MR1*01 as positive controls. Fold increase of Log (CD69 MFI) over vehicle treated C1R (n=3) values are plotted. Data are representative of two experimental repeats.

Supplementary Figure 6 MC.7.G5 clone MR1 sequencing data. Sanger sequencing of the MR1 locus of MC.7.G5 clone analyzed. The MR1*01 allele and MR1*02 allele differ by 1 nucleotide substitution leading to H17R substitution in the MR1*02 allele. The sequences show the MC.7.G5 clone is heterozygous for MR1*01/*02.
