# Supplementary figures and images for: Conserved allomorphs of MR1 drive the specificity of MR1-restricted TCRs
Source: Front Oncol. 2024 Oct 3;14:1419528. doi: 10.3389/fonc.2024.1419528 (PMC11496959; doi:10.3389/fonc.2024.1419528)

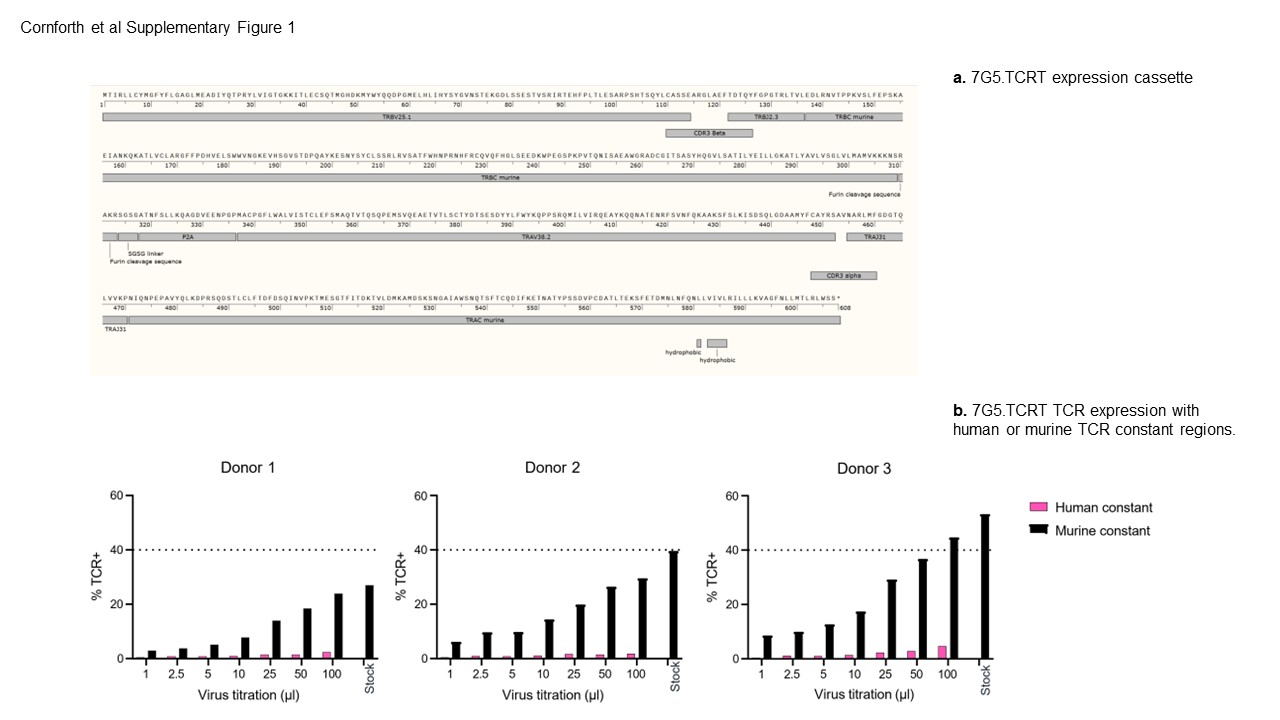

Supplement: Supplementary Figure 1 — 7G5 expression cassette sequence. (A) Map of the 7G5 TCR expression cassette used in this study. Constant alpha and beta regions were murine and hydrophobic residues were introduced into the transmembrane region of the alpha chain as described in the text and denoted on the protein sequence. (B) Comparison of 7G5.TCR-T manufactured with murine or human TCR constant regions. Concentrated lentivirus was titrated using three T cell donors and 7G5 TCR expression, measured by TRBV25 expression (normal expression on only 1% of peripheral T cells). [file Image1.jpeg]

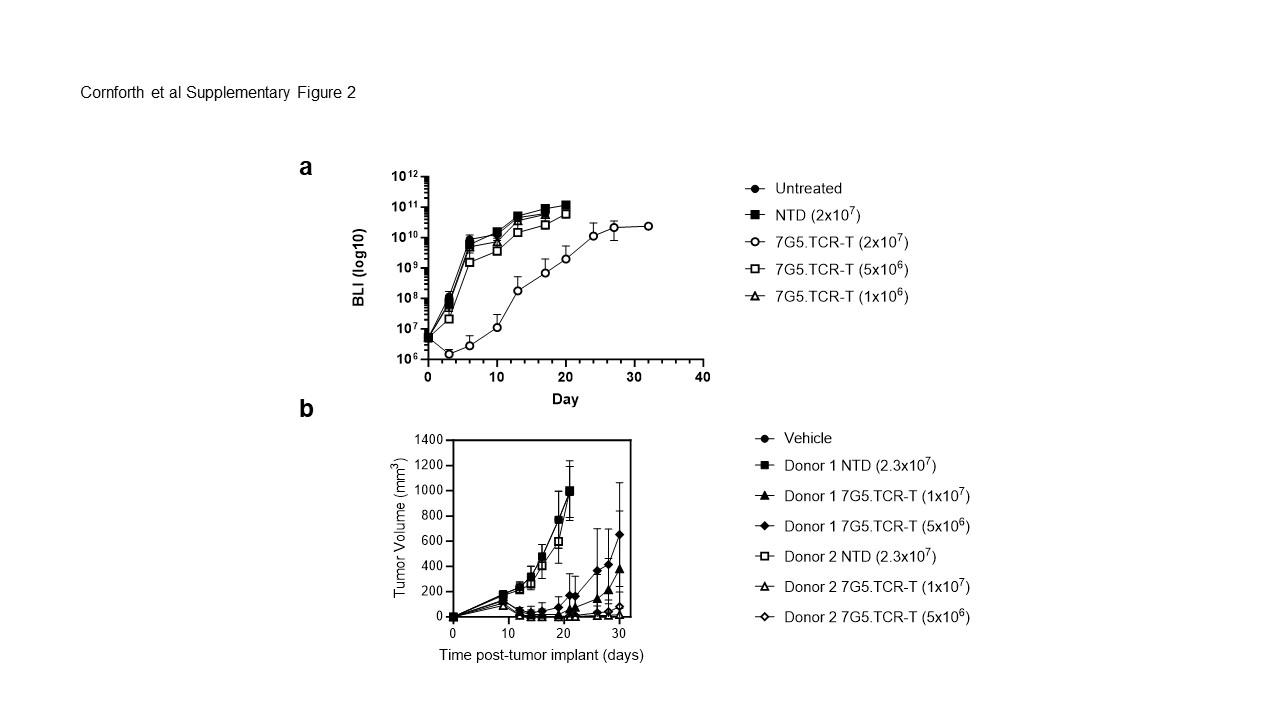

Supplement: Supplementary Figure 2 — 7G5.TCR-T effectiveness in murine tumor models. (A) NSG mice were implanted i.v. with NALM6-Luc tumor cells on day -3 and were then untreated (filled circles) or received 2x10^7^ non-transduced (NTD) T cells (filled squares) or different doses ranging from 2x10^7^-1x10^6^ of 7G5 TCR T cells (open symbols as indicated) from one healthy donor on day 0. Mice were injected i.p with luciferin imaged on day 0, day 3 and twice per week thereafter. In this study the T cells were transduced with a vector that expressed the 7G5 TCR and removed the endogenous TCR using CRISPR-Cas9. See methods section for details of CRISPR-Cas9 methodology. Data are shown as the mean +/-SD (n = 5 mice/group) of whole-body Bioluminescence Imaging (BLI) measurements (photons/second). Multiple student’s t test comparing all time points, untransduced versus 7G5 TCR with a dose of 2x10^7^ shows a significant difference, p<0.005 except at t=0. (B) NSG mice were injected subcutaneously (s.c.) with 5x10^6^ A375-MR1*01 cells. The following day, mice were randomised into groups and on the same day, were injected i.v. with vehicle (filled circles), or T cells from donor 1 or 2 (filled and open symbols). These were 2.3x10^7^ total NTD T cells (squares), or 1x10^7^ (triangles) or 5x10^6^ (diamonds) 7G5 TCR-transduced T cells. In this study the T cells were transduced with a vector that only expressed the 7G5 TCR and did not remove the endogenous TCR. Calliper tumour volume measurements were performed three times a week from Day 5. Data are shown as the mean tumour volume (mm3) +/- SD (n=8 mice/group). ANOVA test with Tukeys post hoc analysis shows a significant difference (p<0.001) between non-transduced T cells and ENA-0001 cells at all doses. [file Image2.jpeg]

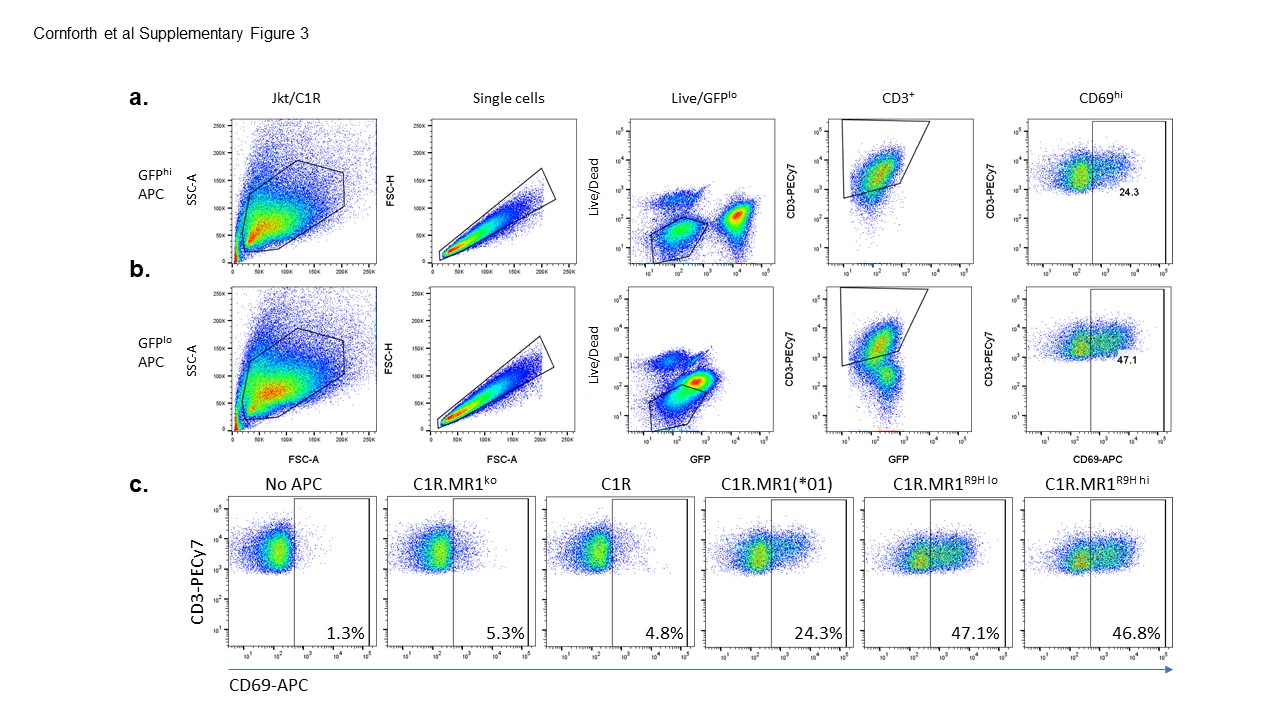

Supplement: Supplementary Figure 3 — Gating strategy for MR1 expression in C1R derivatives. Gating strategy for Jkt.β2mko.7G5 activation assays. Gated for single, live, GFPlo, CD3+ Jkt.β2mko.7G5 cells prior to assessment of CD69 expression. A) Shows example stimulation with C1R.MR1(*01) cells (GFPhi APC). B) Shows example stimulation with C1R.MR1R9Hlo cells (GFPlo APC). C) Example scatter plots of Jkt.β2mko.7G5 CD69 expression on stimulation with different APCs (left to right: No APC, C1R.MR1ko, C1R, C1R.MR1(*01), C1R.MR1R9Hlo, C1R.MR1R9Hhi). [file Image3.jpeg]

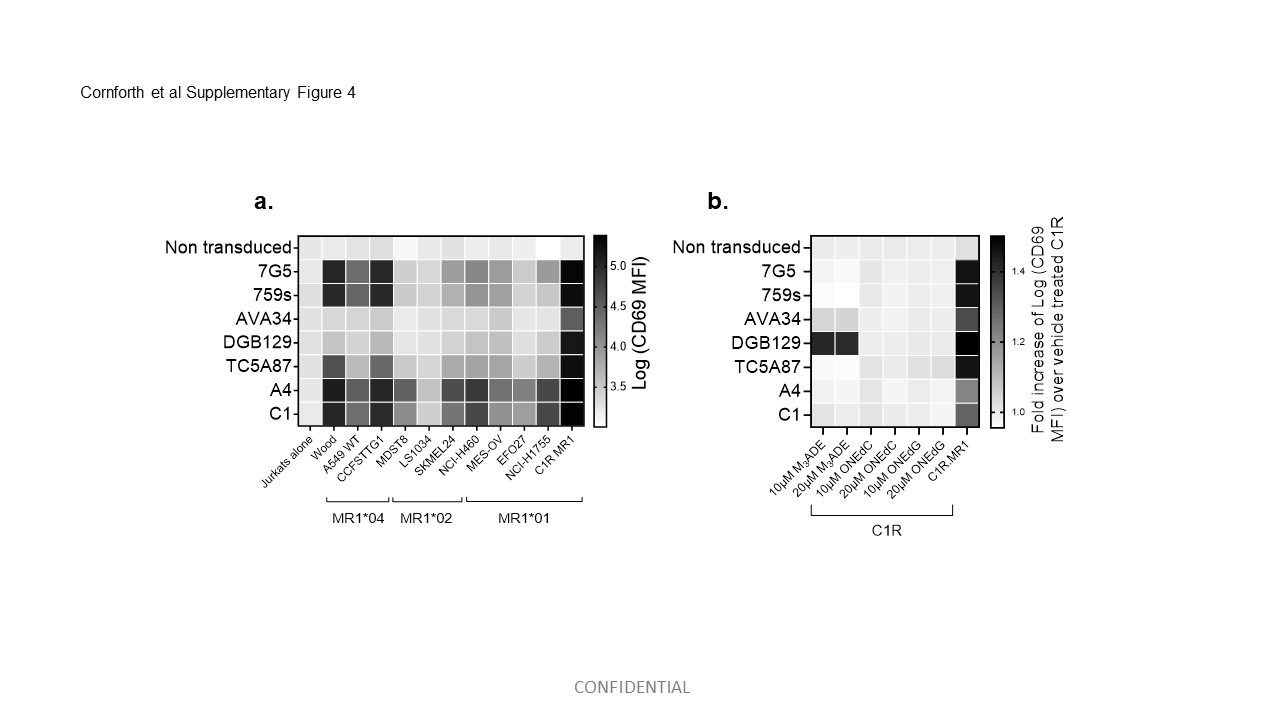

Supplement: Supplementary Figure 4 — 7G5 and 7G5-like TCR-T reactivity against cancer lines expressing MR1*01, MR1*02 and MR1*04 and MR1 ligands. (A) Heatmap showing log median fluorescence intensity values of CD69 surface levels on Jurkat cells expressing one of seven T cell receptors (Y axis) after 24-hour incubation with MR1*01, MR1*02 or MR1*04-expressing cancer cell lines (X axis). Jurkat cells were incubated alone or with C1R cells overexpressing MR1*01 as negative and positive controls respectively. Geometric mean (n=3) values are plotted. Data are representative of two experimental repeats. (B) Heatmap showing log median fluorescence intensity values of CD69 surface levels on Jurkat cells expressing one of seven T cell receptors (Y axis) after 24-hour incubation with C1R cells in the presence of the indicated ligand concentrations (X axis). Jurkat cells were incubated with C1R cells overexpressing MR1*01 as positive controls. Geometric mean (n=3) values are plotted. Data are representative of two experimental repeats. [file Image4.jpeg]

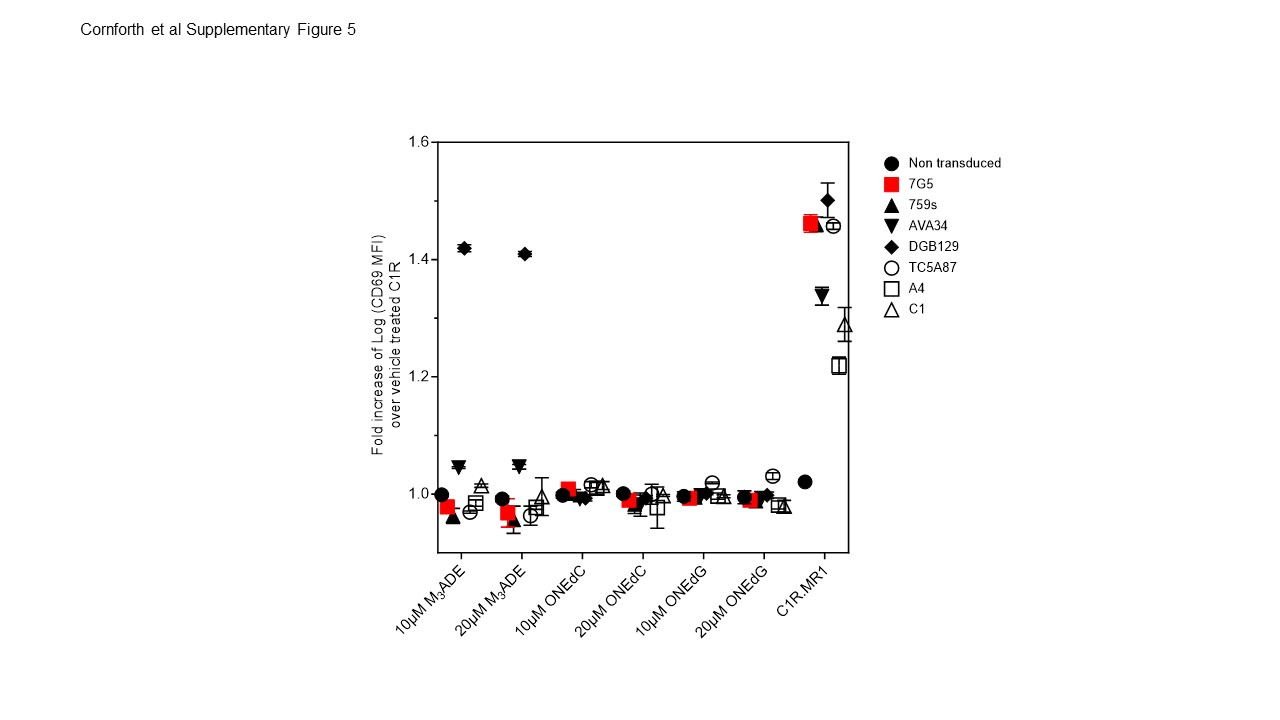

Supplement: Supplementary Figure 5 — 7G5-TCR-T and 7G5-like reactivity to MR1 ligands. Raw data from heat-map represented in Supplementary Figure S4 . Graphical representation of heatmap data presented in Supplementary Figure 4B showing log median fluorescence intensity values of CD69 surface levels on Jurkat cells expressing one of seven T cell receptors (Y axis) after 24-hour incubation with C1R cells in the presence of the indicated ligand concentrations (X axis). C1R cells were incubated with C1R cells overexpressing MR1*01 as positive controls. Fold increase of Log (CD69 MFI) over vehicle treated C1R (n=3) values are plotted. Data are representative of two experimental repeats. [file Image5.jpeg]

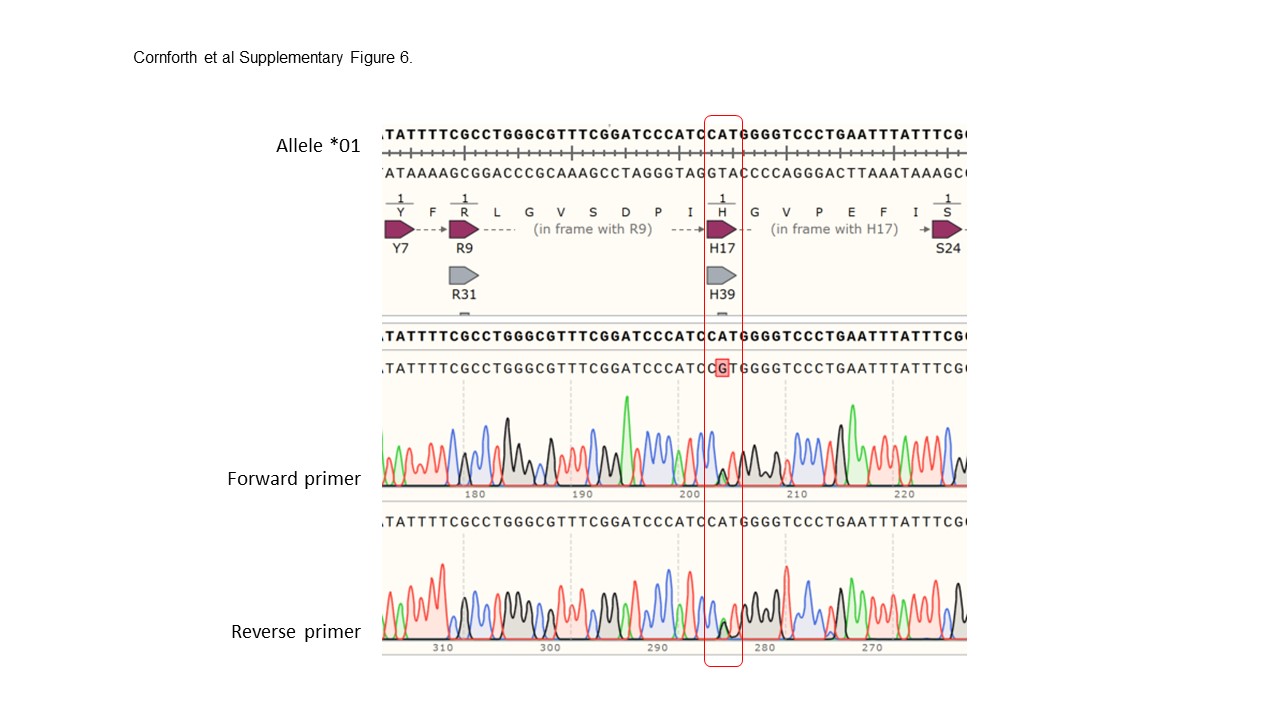

Supplement: Supplementary Figure 6 — MC.7.G5 clone MR1 sequencing data. Sanger sequencing of the MR1 locus of MC.7.G5 clone analyzed. The MR1*01 allele and MR1*02 allele differ by 1 nucleotide substitution leading to H17R substitution in the MR1*02 allele. The sequences show the MC.7.G5 clone is heterozygous for MR1*01/*02. [file Image6.jpeg]
